# Cardiac, oxidative stress, and fibrosis-related biomarkers and new-onset heart failure: Systematic review and meta-analysis

**DOI:** 10.1016/j.ijcrp.2026.200641

**Published:** 2026-04-24

**Authors:** Daniëlle J. Noordermeer, Nicole L.M. de Kruijf, Wichor M. Bramer, Annemien van den Bosch, Maryam Kavousi

**Affiliations:** a Department of Epidemiology, Erasmus MC University Medical Center, Rotterdam, the Netherlands; b Department of Cardiology, Erasmus MC University Medical Center, Rotterdam, the Netherlands; c Medical Library, Erasmus MC University Medical Center, Rotterdam, the Netherlands

**Keywords:** Heart failure, Biomarkers, Cardiac, Oxidative stress, Fibrosis, Healthy volunteers

## Abstract

**Background:**

Circulating biomarkers reflecting pathophysiological pathways, including myocardial stress, injury, oxidative stress, and fibrosis, may facilitate early identification of individuals at increased risk of heart failure (HF) in the general population. However, the strength and consistency of associations between these biomarkers and incident HF have not been comprehensively evaluated.

**Methods:**

A comprehensive search of relevant databases (inception-September 2024) identified population-based studies reporting biomarkers associated with incident HF in individuals without a prior HF history. Pooled hazard ratios (HRs) and 95% confidence intervals (CIs) were calculated using a random effects meta-analysis.

**Results:**

Across 47 included studies, nine biomarkers were significantly associated with new-onset HF in multivariate-adjusted analyses: N-terminal pro b-type natriuretic peptide (NT-proBNP) (HR 2.03, 95% CI: 1.82-2.25, P < 0.01), high-sensitivity cardiac troponin I (hs-cTnI) (HR 1.58, 95% CI: 1.42-1.76, P < 0.01), high-sensitivity cardiac troponin T (hs-cTnT) (HR 1.74, 95% CI: 1.36-2.22, P < 0.01), midregional pro-atrial natriuretic peptide (MR-proANP) (HR 1.54, 95% CI: 1.39-1.71, P < 0.01), b-type natriuretic peptide (BNP) (HR 1.57, 95% CI: 1.44-1.71, P < 0.01), growth differentiation factor 15 (GDF-15) (HR 1.51, 95% CI: 1.36-1.68, P < 0.01), midregional proadrenomedullin (MR-proADM) (HR 1.38, 95% CI: 1.10-1.74, P < 0.01), fibroblast growth factor 23 (FGF-23) (HR 1.17, 95% CI: 1.10-1.25, P < 0.01), and galectin-3 (Gal-3) (HR 1.23, 95% CI: 1.12-1.35, P < 0.01). HF phenotypes and sex differences were also evaluated.

**Conclusions:**

Higher levels of NT-proBNP, hs-cTnI, hs-cTnT, MR-proANP, BNP, GDF-15, MR-proADM, FGF-23, and Gal-3 were associated with a larger risk of incident HF, supporting their utility as biomarkers for predicting HF development.

## Highlights

• First meta-analysis of cardiac, oxidative stress, and fibrosis biomarkers for incident heart failure (HF).
• Nine circulating biomarkers independently predict new-onset HF.
• Natriuretic peptides and troponins show the strongest associations with HF risk.
• Fibrosis and oxidative stress biomarkers add prognostic value beyond cardiac markers.
• Sex- and phenotype-specific differences suggest potential for personalized HF risk prediction.

## Graphical abstract

Biomarkers Associated with New-Onset Heart Failure in the General Population. *CI, confidence interval; HR, hazard ratio.*

**Figure:**
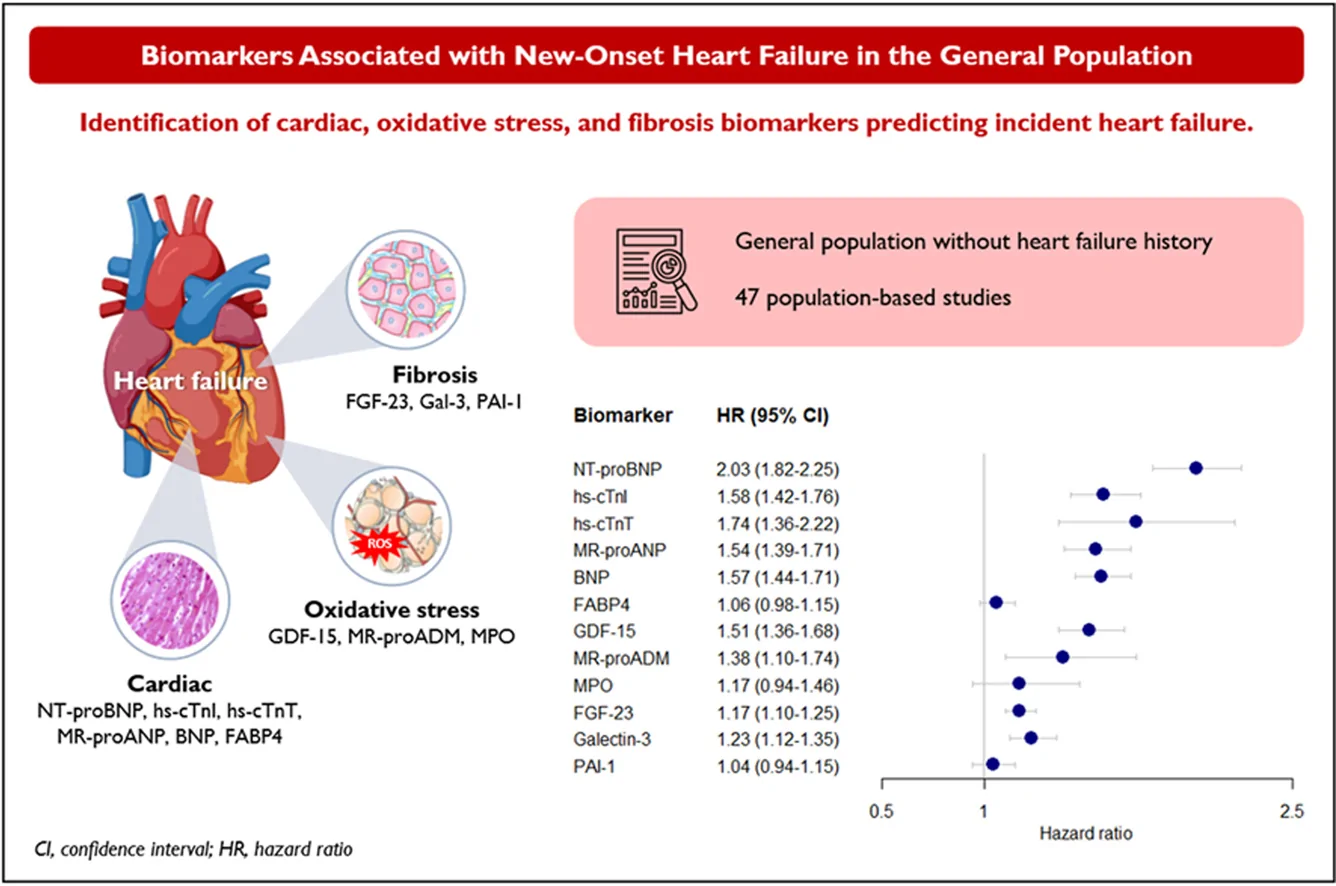

## 1 Introduction

Heart failure (HF) affects an estimated 56 million people globally, with an incidence of approximately 5 per 1000 person-years in adults, leading to high morbidity and mortality [[1], [2], [3]]. HF arises from complex mechanisms, including cardiac injury, oxidative stress, fibrosis, inflammation, renal dysfunction, neurohormonal activation, and altered adipose tissue metabolism [4].

Circulating biomarkers provide insights into these pathways. In this review, we focus on three key pathophysiological processes—cardiac injury, oxidative stress, and fibrosis—while acknowledging that other mechanisms also contribute significantly to HF development. Cardiac biomarkers like natriuretic peptides (NPs) and troponins reflect myocardial stretch, chamber dilatation, and myocardial injury [[5], [6], [7]]. Oxidative stress biomarkers emerge from deregulated reactive oxygen (ROS) and nitrogen species (RNS), disrupting cardiac energy metabolism and causing overload [8]. Fibrosis biomarkers indicate excessive extracellular matrix deposition, leading to myocardial stiffness and impaired function. Importantly, some biomarkers participate in multiple pathophysiological processes, underscoring the interconnected nature of HF biology.

Despite extensive research, the predictive value of many biomarkers for new-onset HF in the general population remains uncertain. Furthermore, while HF pathophysiology differs between men and women, the sex-specific predictive value of biomarkers is unclear.

To address these gaps, we conducted the first systematic review and meta-analysis of cardiac, oxidative stress, and fibrosis biomarkers in relation to new-onset HF, evaluating their predictive value and potential sex differences. This analysis aims to provide a better understanding of how these biomarkers can enhance HF prediction and management.

## 2 Methods

This systematic review and meta-analysis adhere to the PRISMA and MOOSE guidelines, with the protocol registered in PROSPERO (CRD42023422963).

### 2.1 Data availability statement

All data analyzed in this study were obtained from previously published sources. No new data were generated.

### 2.2 Search strategy and study selection

An experienced information specialist (W.B.) developed a comprehensive search strategy, executed in Medline Ovid, Embase.com, Web of Science Core Collection and Cochrane Central Register of Controlled Trials in February 2023, with the last update on September 19, 2024. Search terms included 1) heart or cardiac failure, 2) biomarkers (e.g., BNP, troponin), 3) incidence or epidemiology, and 4) population or health volunteers. As part of the search strategy, non-human, cell/tissue studies, case reports, congress, abstract, book, chapters were excluded. Full search strategies are available in Supplementary Methods.

References were imported into EndNote, and duplicates were removed by the medical librarian. Two reviewers (D.N. and N.K.) independently screened titles and abstract, resolving discrepancies through discussion or consultation with a third reviewer (M.K.). The same procedure was applied for full-text screening. Study quality was assessed using the Newcastle-Ottawa criteria.

### 2.3 Selection criteria

Eligible studies investigated cardiac, oxidative stress, or fibrosis biomarkers in population-based cohorts (≥18 years) assessing new-onset HF. Reviews, meta-analyses, non-English full-text studies, and studies including prevalent HF cases were excluded. To ensure specificity to incident HF, studies that primarily investigated other cardiovascular diseases (e.g., myocardial infarction, valvular disease, or congenital heart disease) without clear reporting on new-onset HF were also excluded. To prevent duplicate data, for multiple studies investigating the same biomarker within the same study populations, the study with the largest sample size was included; if sample sizes were equal, the most recent publication was selected. Biomarkers analyzed in only two independent studies were excluded from the meta-analysis but reported in the results. All included studies adhered to the Declaration of Helsinki.

### 2.4 Data extraction

The following data were extracted manually: first author, year, cohort name, visit, follow-up duration, number of participants, sex distribution, incident HF cases, mean age, biomarker hazard ratios (HRs) with adjustments. Authors of studies presenting HRs categorically (e.g., tertiles or quartiles) were contacted to request continuous HRs.

### 2.5 Statistical analyses

Pooled HRs and 95% confidence intervals (CIs) were calculated using the generic inverse variance method, incorporating the most extensively adjusted model from each study. Analyses were stratified by sex, and HF with preserved ejection fraction (HFpEF) and HF with reduced ejection fraction (HFrEF). Sensitivity analyses were performed by grouping the analyses into simple multivariate models (adjusted for age and sex), and complex multivariate models (additionally adjusted for cardiovascular risk factors). Further sensitivity analyses were performed on logarithmic and non-transformed HRs. Additionally, sensitivity analyses accounted for study quality (poor, fair).

A random-effect model was used due to expected heterogeneity, assessed by I^2^ statistics. If I^2^ exceeded 75%, additional sensitivity analyses explored heterogeneity sources. Publication bias was evaluated via funnel plots. Statistical significance was considered at a two-tailed P-value ≤0.050. Analyses were performed in IBM SPSS Statistics (v25.0, IBM Corp., Armonk, New York, USA) and R (v4.0.3, R Foundation, Vienna, Austria).

## 3 Results

**Fig. 1:**
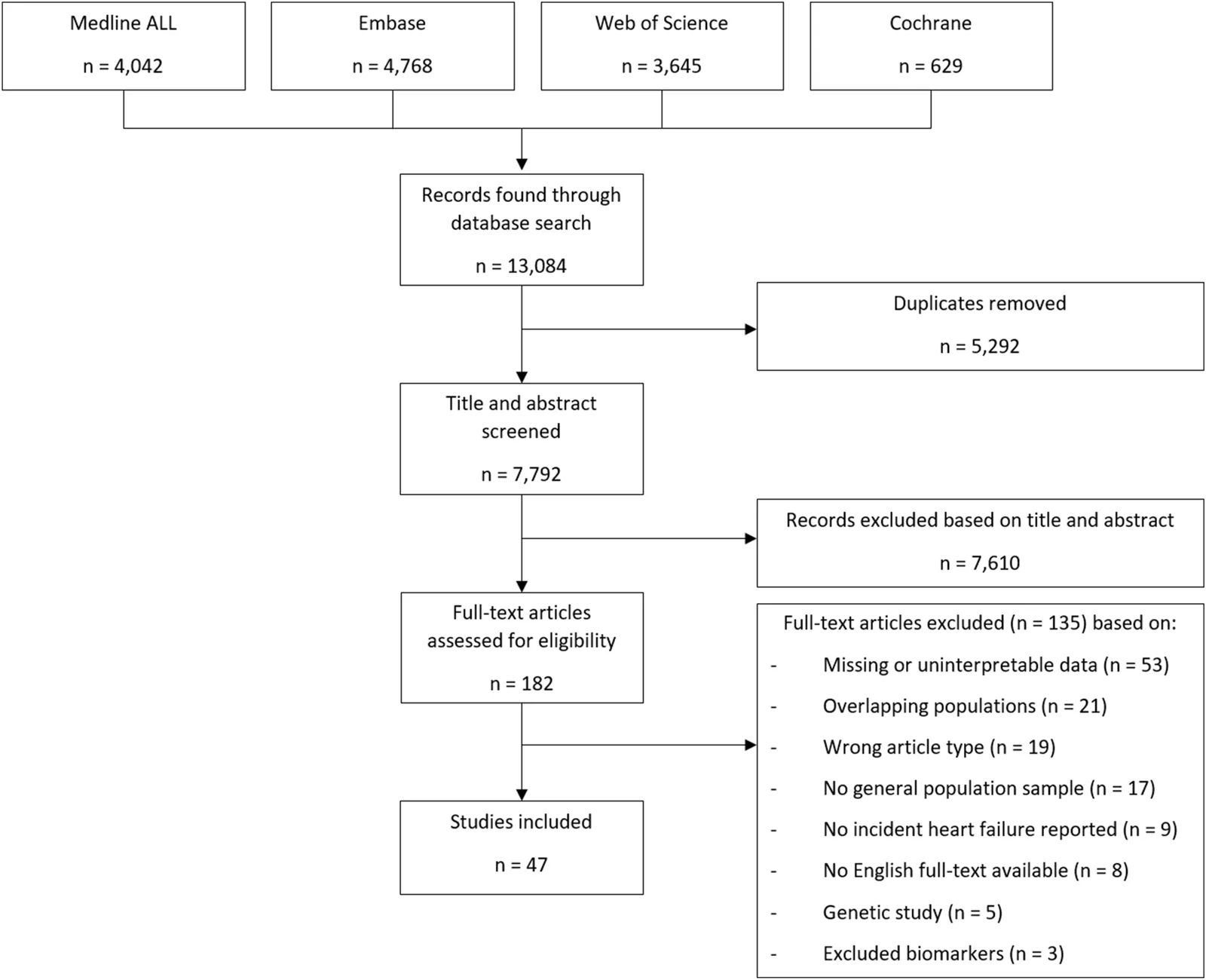
**Study Selection Flowchart.** The initial search yielded 13,084 references across four databases. After removing 5292 duplicates, 7792 unique records were screened by title and abstract. Of these, 182 articles (2.3%) underwent full-text review. Full-text exclusions were due to: missing or uninterpretable data (n = 53), overlapping populations (n = 21), ineligible article types (n = 19), non-general population samples (n = 17), absence of incident heart failure data (n = 9), non-English full texts (n = 8), genetic study designs (n = 5), and biomarkers investigated in only a single study (n = 3). A total of 47 studies were included in the final meta-analysis. *n, number.*

The initial search yielded 13,084 references across four databases. After removing 5292 duplicates, 7792 unique records were screened by title and abstract. Following title and abstract screening, 182 (2.3%) manuscripts were suitable for full-text screening. Studies were excluded for the following reasons: missing or uninterpretable data (n = 53), overlapping populations (n = 21), incorrect article type (n = 19), non-general population studies (n = 17), not investigating HF incidence (n = 9), no available English full-text (n = 8), genetic studies (n = 5), and biomarkers reported in only one study (n = 3). Ultimately, 47 manuscripts remained eligible for the meta-analysis. A detailed overview study selection process is presented in Fig. 1, with study characteristics summarized in Table A.1. An overview of biomarkers associations with incident HF is provided in Table 1.

**Table 1:** Summary of biomarker associations with incident heart failure: Meta-analysis.

Biomarkers	HR (95% CI)	p-value	Heterogeneity	p-value	Number of studies	Total N	HF events
**Cardiac**							
NT-proBNP	2.03 (1.82; 2.25)	<0.001	89%	<0.001	13	109,497	6156
hs-cTnI	1.58 (1.42; 1.76)	<0.001	86%	<0.001	10	100,878	4180
hs-cTnT	1.74 (1.36; 2.22)	<0.001	97%	<0.001	6	53,788	2592
MR-proANP	1.54 (1.39; 1.71)	<0.001	21%	0.281	3	21,603	991
BNP	1.57 (1.44; 1.71)	<0.001	0%	0.929	3	12,477	773
FABP4	1.06 (0.98; 1.15)	0.167	0%	0.424	3	7559	1482
**Oxidative stress**							
GDF-15	1.51 (1.36; 1.68)	<0.001	58%	0.051	5	22,892	2987
MR-proADM	1.38 (1.10; 1.74)	0.005	82%	0.004	3	21,603	991
MPO	1.17 (0.94; 1.46)	0.161	87%	<0.001	3	8721	958
**Fibrosis**							
FGF-23	1.17 (1.10; 1.25)	<0.001	43%	0.092	8	34,779	2813
Gal-3	1.23 (1.12; 1.35)	<0.001	64%	0.004	8	32,759	2016
PAI-1	1.04 (0.94; 1.15)	0.475	0%	0.665	3	13,438	713

BNP, b-type natriuretic peptide; FABP4, fatty acid binding protein 4; FGF-23, fibroblast growth factor 23; HF, heart failure; HR, hazard ratio; Gal-3, galectin-3; GDF-15, growth differentiation factor 15; hs-cTnI, high-sensitivity cardiac troponin I; hs-cTnT, high-sensitivity cardiac troponin T; MPO, myeloperoxidase; MR-proADM, midregional proadrenomedullin; MR-proANP, midregional pro-atrial natriuretic peptide; N, number; NT-proBNP, N-terminal pro b-type natriuretic peptide; PAI-1, plasminogen activator inhibitor-1.

### 3.1 Cardiac biomarkers and new-onset HF

Thirteen articles (n = 109,497, HF events = 6156) evaluated N-terminal pro b-type natriuretic peptide (NT-proBNP) and its association with incident HF. Pooled analysis showed a significant association (HR 2.03, 95% CI 1.82-2.25) (Figure A.1). Sensitivity analyses excluding Brouwers et al., Funke-Kaiser et al., and Segar et al. yielded similar results. Despite further sensitivity testing, high heterogeneity persisted, with Harris et al. as a clear outlier. Excluding this study did not significantly alter heterogeneity. Despite meticulous review of the studies, no clear explanation for the high heterogeneity was identified.

For high-sensitivity cardiac troponin I (hs-cTnI), ten studies (n = 100,878, HF events = 4180) confirmed a significant association with incident HF (HR 1.58, 95% CI 1.42-1.76). In simple multivariate models, hs-cTnI remained significantly associated (HR 1.62, 95% CI 1.52-1.72). Sensitivity analysis excluding the two oldest studies (Neumann et al. and Wang et al.) reduced heterogeneity from 86% to 72% (Figure A.2).

Six studies (n = 53,788, HF events = 2592) examined high-sensitivity cardiac troponin T (hs-cTnT), revealing a significant association with HF (HR 1.74, 95% CI 1.36-2.22), also in logarithmic HRs (Figure A.2). Excluding two outlier studies reduced heterogeneity from 97% to 56% (Figure A.2). Despite a thorough review, no further explanation for the remaining heterogeneity was found.

Three studies (n = 21,603, HF events = 991) found a significant association of midregional pro-atrial natriuretic peptide (MR-proANP) and incident HF (HR 1.54, 95% CI 1.39-1.71).

Three studies (n = 12,477, HF events = 773) also showed a significant association of BNP with HF (HR 1.57, 95% CI 1.44-1.71).

For fatty-acid binding protein 4 (FABP4), three studies (n = 7,559, HF events = 1482) showed no significant association with HF (HR 1.06, 95% CI 0.98-1.15). However, in simple multivariate models, FABP4 was associated with HF (HR 1.34, 95% CI 1.27-1.42) (Figure A.2).

### 3.2 Oxidative stress-related biomarkers in association with new-onset HF

Five studies (n = 22,892, HF events = 2987) investigated growth differentiation factor 15 (GDF-15) (Figure A.3), confirming a significant association (HR 1.51, 95% CI 1.36-1.68).

Midregional proadrenomedullin (MR-proADM) was investigated in three studies (n = 21,603, HF events = 991), revealing a significant association (HR 1.38, 95% CI 1.10-1.74).

Three studies (n = 8,721, HF events = 958) found no significant association between myeloperoxidase (MPO) and HF (HR 1.17, 95% CI 0.94-1.46).

### 3.3 Fibrosis biomarkers in association with new-onset HF

Fibroblast growth factor 23 (FGF-23) was associated with HF (HR 1.17, 95% CI 1.10-1.25), based on eight studies (n = 34,779, HF events = 2813) (Figure A.4). Additional sensitivity analyses were conducted due to the moderate quality of the study by Sharma et al., but similar results were found.

Galectin-3 (Gal-3) was associated with HF (HR 1.23, 95% CI 1.12-1.35), after pooling data from eight studies (n = 32,759, HF events = 2016). A similar association was observed in simple multivariate models (HR 1.33, 95% CI 1.18-1.49) across five studies.

Three studies (n = 13,438, HF events = 713) found no association between plasminogen activator inhibitor-1 (PAI-1) and HF (HR 1.04, 95% CI 0.94-1.15).

### 3.4 Biomarkers investigated in two studies

Certain biomarkers, including cardiac troponin T (cTnT), insulin-like growth factor 1 (IGF-1), and insulin-like growth factor-binding protein 1 and 2 (IGFBP-1, IGFBP-2), were analyzed in only two studies, preventing meta-analysis. Elevated cTnT levels were linked to increased HF risk (Suthahar et al.: HR 1.51, 95% CI 1.32-1.72; deFilippi et al.: HR 1.44, 95% CI 1.33-1.55) [9,10]. No association was found for IGF-1 (Kaplan et al.: HR 1.12, 95% CI 0.99-1.26; Ho et al.: HR 0.91, 95% CI 0.80-1.04). IGFBP-1 was associated with incident HF (Kaplan et al.: HR 1.22, 95% CI 1.07-1.39; Ho et al.: HR 1.37, 95% CI 1.17-1.61), while IGFBP-2 showed conflicting results (Molvin et al.: HR 1.01, 95% CI 0.84-1.22; Ho et al.: HR 1.30, 95% CI 1.11-1.51) [[11], [12], [13]].

### 3.5 Sex-specific findings in the association of biomarkers with new-onset HF

Sex-related differences were observed in biomarkers associations with new-onset HF (Table 2). In one study, participants were stratified into two risk groups based on the presence or absence or prior cardiovascular history, referred to as high-risk and low-risk, respectively [14]. Prior cardiovascular history was defined as previous hospitalization for myocardial infarction or cerebrovascular accident, or the use of antihypertensive, lipid-lowering, or glucose-lowering medications at baseline assessment. NT-proBNP showed a stronger association with new-onset HF in low-risk women (HR 2.33, 95% CI 1.43-3.80) than in low-risk men (HR 1.35, 95% CI 1.00-1.82). In contrast, among high-risk individuals, the association was stronger in men, a pattern also observed with MR-proANP and MR-proADM [14]. Hs-cTnT showed a greater HR in high-risk women (HR 1.87, 95% CI 1.44-2.43) than in men (HR 1.59, 95% CI 1.33-1.89). However, overall, both hs-cTnI and hs-cTnT were more strongly associated with new-onset HF in men. Gal-3 showed stronger associations with new-onset HF in high-risk men compared to women, while results in low-risk groups and another cohort showed minimal sex differences. PAI-1 demonstrated modest and similar associations in both sexes, with no clear sex-specific pattern.

**Table 2:** Meta-analysis of biomarkers in association with new-onset heart failure, stratified by sex.

Biomarker	Author		Women	Men
NT-proBNP	Brouwers	Low risk group	2.33 (1.43-3.80)	1.35 (1.00-1.82)
		High risk group	2.20 (1.34-3.62)	3.21 (2.47-4.17)
	Magnussen		1.54 (1.37-2.05)	1.89 (1.75-2.05)
	Zhu^2^		1.89 (1.69-2.10)	1.91 (1.68-2.17)
Hs-cTnI	Neumann		1.12 (0.98-1.27)	1.24 (1.12-1.38)
	Zhu^1^		1.34 (1.09-1.66)	1.58 (1.24-2.02)
Hs-cTnT	Brouwers	Low risk group	1.38 (0.95-2.00)	1.38 (1.04-1.82)
		High risk group	1.87 (1.44-2.43)	1.59 (1.33-1.89)
	Zhu^2^		1.50 (1.32-1.70)	1.69 (1.46-1.95)
MR-proANP	Brouwers	Low risk group	1.04 (0.74-1.46)	1.06 (0.74-1.53)
		High risk group	2.16 (1.22-3.83)	2.48 (1.89-3.27)
MR-proADM	Brouwers	Low risk group	0.62 (0.43-0.90)	1.01 (0.70-1.47)
		High risk group	1.35 (1.02-1.80)	1.46 (1.25-1.71)
Gal-3	Brouwers	Low risk group	0.89 (0.60-1.32)	0.93 (0.68-1.28)
		High risk group	1.17 (0.93-1.47)	1.42 (1.15-1.76)
	Jagodzinski		1.13 (0.98-1.29)	1.08 (0.96-1.22)
PAI-1	Suthahar		1.11 (0.93-1.31)	1.09 (0.95-1.25)

Gal-3, galectin-3; hs-cTnI, high-sensitivity cardiac troponin I; hs-cTnT, high-sensitivity cardiac troponin T; MR-proADM, midregional proadrenomedullin; MR-proANP, midregional pro-atrial natriuretic peptide; NT-proBNP, N-terminal pro b-type natriuretic peptide; PAI-1, plasminogen activator inhibitor-1.

### 3.6 HF with preserved versus reduced ejection fraction

Several studies distinguished HF phenotypes (Table A.2). NT-proBNP exhibited a higher HR in HFrEF compared to HFpEF, a pattern also observed in hs-cTnT, MR-proADM, and Gal-3. MR-proANP was more strongly associated with HFrEF in high-risk individuals (HFrEF: HR 2.96, 95% CI 2.07-4.21; HFpEF: HR 1.49, 95% CI 0.92-2.40). FGF-23 yielded conflicting results, with one study favoring HFrEF and another HFpEF. Lastly, hs-cTnI had comparable HRs for both HF subtypes.

## 4 Discussion

This meta-analysis identified significant associations between biomarkers related to cardiac function, oxidative stress, and fibrosis with new-onset HF, as visually summarized in the graphical abstract. Cardiac biomarkers (NT-proBNP, hs-cTnI, hs-cTnT, MR-proANP, BNP), oxidative stress markers (GDF-15, MR-proADM), and fibrosis-related proteins (FGF-23, Gal-3) associated with incident HF after adjusting for traditional risk factors. Notably, sex-related differences emerged in these associations, suggesting potential implications for personalized HF risk stratification.

### 4.1 Current guidelines for screening HF biomarkers

NPs, including BNP and NT-proBNP, are widely recommended for HF diagnosis and risk stratification. The ACC/AHA/HFSA guidelines advocate their use for early HF detection and management, while NICE guidelines recommend NT-proBNP screening in suspected HF cases [[15], [16], [17]].

### 4.2 Cardiac biomarkers and new-onset HF

Cardiac biomarkers play key roles in HF development. NT-proBNP, a key marker of myocardial stretch, remains one of the strongest predictors of HF, despite high heterogeneity across studies. BNP and MR-proANP were also significantly associated with HF risk, with lower variability across studies.

Troponins (hs-cTnI, hs-cTnT), markers of myocardial injury, are crucial for HF risk prediction [7]. Despite high heterogeneity, these biomarkers showed strong associations with HF. When evaluated in combination with NT-proBNP (Table A.3), mutual adjustment generally attenuated, but did not eliminate the associations, indicating that these biomarkers share overlapping yet partly distinct prognostic information [14,[18], [19], [20], [21], [22]]. This is biologically plausible, as NPs primarily reflect myocardial stretch, whereas troponins capture subclinical myocardial injury. These findings support the concept of a multimarker approach; however, the observed attenuation suggests that the incremental value of combining biomarkers may be modest and context-dependent. Importantly, our analysis was not designed to assess improvements in discrimination. Therefore, routine use of combined biomarker panels for HF risk prediction cannot yet be recommended. FABP4, another cardiac pathway biomarker, showed no association with HF.

In summary, cardiac biomarkers such as NT-proBNP, hs-cTnI, hs-cTnT, MR-proANP, and BNP demonstrate strong associations with incident HF. While NPs and troponins exhibited the highest HRs, heterogeneity across studies necessitates cautious interpretation.

### 4.3 Oxidative stress-related biomarkers and new-onset HF

Oxidative stress contributes significantly to HF pathogenesis, driven by an overproduction of ROS and RNS [8]. MR-proADM, which maintains endothelial barrier function, and GDF-15, expressed in response to cellular stress, were both associated with new-onset HF [[23], [24], [25], [26]]. However, MR-proADM's association showed high heterogeneity and was examined in only three studies. MPO, a marker of vascular inflammation and oxidative stress, showed no evidence of association with HF.

### 4.4 Fibrosis biomarkers and new-onset HF

Fibrosis, characterized by excessive extracellular matrix deposition, is important in cardiac remodeling leading to HF. Gal-3, a macrophage-derived mediator of fibrosis, was strongly associated with HF risk, as FGF-23, a regulator of cellular proliferation [[27], [28], [29], [30]]. PAI-1, which inhibits matrix breakdown, was not significantly linked to HF [31,32]. Overall, fibrosis biomarkers like Gal-3 and FGF-23 demonstrated low heterogeneity, suggesting consistent associations across studies.

### 4.5 Sex-specific implications

Sex-based differences in HF biomarkers reflect variations in HF phenotypes and pathology [33,34]. Men are more likely to develop HFrEF, while women are at higher risk for HFpEF, which often occurs later and with non-ischemic etiologies [35]. However, sex-specific analyses yielded inconsistent results, limiting the ability to perform robust meta-analyses. This underscores the need for further research to explore sex-specific impacts of biomarkers in HF risk prediction.

### 4.6 HF phenotypes: preserved vs. reduced ejection fraction

HFpEF and HFrEF exhibit distinct epidemiology and molecular pathways [[36], [37], [38]]. HFpEF is associated with systemic inflammation and endothelial dysfunction, while HFrEF involves cardiomyocytes loss and neurohormonal activation [[39], [40], [41]]. Biomarkers like NT-proBNP, hs-cTnT, MR-proANP, MR-proADM, and Gal-3 differ in their associations with these phenotypes. However, most studies included in this analysis did not differentiate between HFpEF and HFrEF, limiting the ability to conduct phenotype-specific meta-analyses.

### 4.7 Clinical implications and integrated risk assessment

In clinical practice, prediction and diagnosis of new-onset HF cannot rely solely on circulating biomarkers. A comprehensive clinical assessment remains essential, including evaluation of symptoms and signs, pre-existing cardiovascular and non-cardiovascular diseases, family history, and lifestyle factors, as well as complementary diagnostic investigations such as imaging (e.g., echocardiography) [15,42,43]. This integrated approach aligns with current clinical frameworks emphasizing multimodal risk assessment for HF prevention and early detection.

### 4.8 Strengths and limitations

This meta-analysis provides a comprehensive overview of biomarkers involved in cardiac, oxidative stress, and fibrosis-related pathways, and their association with new-onset HF. A key strength is that all included studies were prospective or retrospective population-based cohort studies, providing robust longitudinal data. The inclusion of multiple biomarker classes also allows for a broader exploration of pathophysiological mechanisms underlying HF development.

However, several limitations should be considered when interpreting the results. First, all included studies originated from Europe, United States, Australia, or New Zealand. Comparisons between black and white participants were limited to a few studies [[44], [45], [46]], which may affect the generalizability of our findings. In addition, six out of thirteen studies with initially uninterpretable data could not provide useable information, leading to their exclusion. Substantial heterogeneity was observed across studies for specific biomarkers for several biomarkers, including NT-proBNP, hs-cTnI, hs-cTnT, MR-proADM, and MPO (Figure A.5 and A.6). While funnel plots were used to assess potential publication bias, their interpretation is limited by the small number of studies per biomarker [47].

Regarding the heterogeneity in NT-proBNP and hs-cTnT, a comprehensive analysis of the studies did not yield a clear explanation. We examined possible sources of heterogeneity, including age, geographic region, and follow-up duration, but these factors did not explain the variability observed. Elevated levels of these biomarkers may occur in individuals with diverse comorbidities and are not specific to HF [48,49], reflecting their broader prognostic roles. Although GDF-15 and MR-proADM are not direct indicators of oxidative stress, they were included in this category due to their established links to systemic stress and redox-sensitive pathways relevant to HF. We acknowledge that many biomarkers, including FABP4, MR-proADM, and GDF-15, are pleiotropic and may also reflect processes such as inflammation, fibrosis, or metabolic dysfunction. This overlap highlights the complexity of HF pathophysiology and the challenge of assigning biomarkers to discrete biological pathways. Furthermore, although we evaluated associations of biomarkers with HF phenotypes (HFpEF vs HFrEF) and sex-specific differences, most included studies did not consistently report stratified results, limiting the ability to conduct robust meta-analyses for these subgroups. This highlights a need for future research to better understand phenotype- and sex-specific biomarker associations in HF risk prediction.

### 4.9 Conclusions

This meta-analysis offers a comprehensive overview of the associations between cardiac, oxidative stress, and fibrosis-related biomarkers and new-onset HF. Among cardiac biomarkers, NPs and troponins exhibit the highest HRs, reflecting strong association with HF risk, despite high heterogeneity among studies. MR-proANP and BNP have slightly lower HRs with lower heterogeneity. Regarding oxidative stress biomarkers, GDF-15 and MR-proADM are associated with new-onset HF. Fibrosis biomarkers such as Gal-3 and FGF-23 also hold potential for HF risk stratification. Additionally, our study underscores the need for sex-specific approaches to HF risk prediction. Further research is warranted to provide robust evidence before establishing widespread clinical recommendations for these biomarkers in HF risk assessment in the general population.

## Funding

This work was supported by Senior Scientist Grant 03-004-2021-T050 from the Dutch Heart Foundation and the Innovative Health Initiative Joint Undertaking (IHI JU) and 10.13039/100032555Breakthrough T1D under grant agreement No 101112022 (https://icare4cvd.eu/). The JU receives support from the European Union’s Horizon Europe research and innovation program and COCIR, EFPIA, Vaccines Europe, EuropaBio and MedTech Europe.

## CRediT authorship contribution statement

**Daniëlle J. Noordermeer:** Conceptualization, Data curation, Formal analysis, Investigation, Methodology, Project administration, Validation, Visualization, Writing – original draft, Writing – review & editing. **Nicole L.M. de Kruijf:** Conceptualization, Investigation, Methodology, Writing – review & editing. **Wichor M. Bramer:** Data curation, Formal analysis, Resources, Software, Validation. **Annemien van den Bosch:** Conceptualization, Formal analysis, Supervision, Writing – review & editing. **Maryam Kavousi:** Conceptualization, Formal analysis, Funding acquisition, Methodology, Resources, Software, Supervision.

## Declaration of competing interest

The authors declare no competing interests. The funders had no role in study design, data collection and analysis, decision to publish, or preparation of the manuscript.
