# Supplementary material for: Cardiac, oxidative stress, and fibrosis-related biomarkers and new-onset heart failure: Systematic review and meta-analysis
Source: Int J Cardiol Cardiovasc Risk Prev. 2026 Apr 24;30:200641. doi: 10.1016/j.ijcrp.2026.200641 (PMC13534987; doi:10.1016/j.ijcrp.2026.200641)
Supplement: Supplementary file 1 [file mmc1.pdf]

Erasmus MC

Supplementary data

Contents

Supplemental Methods 2

Search Strategy 2

Additional Search 8

Supplemental Figures 15

Figure A.1. 17

Figure A.2. 21

Figure A.3. 23

Supplemental Tables 24

Table A.1 24

Table A.2 32

Table A.3 33

Supplemental Methods

Search Strategy

Database searched	Platform	Years of coverage	Records	Records after duplicates removed
Medline ALL	Ovid	1946 - Present	4042	4028
Embase	Embase.com	1971 - Present	4768	2065
Web of Science Core Collection*	Web of Knowledge	1975 - Present	3645	1301
Cochrane Central Register of Controlled Trials**	Wiley	1992 - Present	629	398
**Total**			**13084**	**7792**

*Science Citation Index Expanded (1975-present) ; Social Sciences Citation Index (1975-present) ; Arts & Humanities Citation Index (1975-present) ; Conference Proceedings Citation Index- Science (1990-present) ; Conference Proceedings Citation Index- Social Science & Humanities (1990-present) ; Emerging Sources Citation Index (2005-present)

** Manually deleted abstracts from trial registries

No other database limits were used than those specified in the search strategies

Medline ALL OVID (1946-)

(exp Heart Failure / OR (((heart OR cardiac OR cardial OR myocard* OR cardis OR ventric*) ADJ3 (failure* OR insufficien* OR decompensat* OR incompeten* OR dysfunction*))).ab,ti,kw.) AND (exp Biomarkers/ OR exp Natriuretic Agents/ OR Atrial Natriuretic Factor/ OR exp Natriuretic Peptide, Brain/ OR exp Troponin/ OR exp Fatty Acid-

Binding Proteins/ OR Myoglobin/ OR exp Growth Differentiation Factors/ OR Peroxidase/ OR Ceruloplasmin/ OR Adrenomedullin / OR Malondialdehyde/ OR exp Trypsin Inhibitors/ OR alpha 1-Antitrypsin/ OR Scavenger Receptors, Class E/ OR Oxidative Stress/ OR Galectin 3/ OR exp Matrix Metalloproteinases/ OR Fibroblast Growth Factor-23/ OR exp Insulin-Like Growth Factor Binding Proteins/ OR Syndecan-1/ OR Transforming Growth Factor beta / OR exp Tissue Inhibitor of Metalloproteinases/ OR ((biological* ADJ3 marker*) OR biomarker* OR (cardiac* ADJ3 biological* ADJ3 marker*) OR ((cardiac* OR cardiovasc*) ADJ3 (biomarker* OR bio*-marker*)) OR (natriuretic ADJ3 (factor* OR peptide* OR hormone*)) OR (troponin*) OR probnp OR ntprobnp OR bnp OR tnt OR (fatty-acid ADJ3 binding ADJ3 protein*) OR fabp OR myoglobin* OR (growth ADJ3 differentiat* ADJ3 factor*) OR gdf OR myeloperoxidas* OR ceruloplasmin* OR proadrenomedullin* OR proadm* OR malonaldehyd* OR malondialdehyd* OR (trypsin ADJ3 inhibitor*) OR ((alpha-1 OR a-1 OR a1) ADJ3 protein*) OR (oxidi* ADJ3 (low-density-lipoprotein OR ldl) ADJ3 receptor*) OR (oxidative ADJ3 stress*) OR galectin-3 OR gal-3 OR galectin3 OR gal3 OR gelatinase-A OR stromelysin* OR (neutrophil ADJ3 collagenas*) OR gelatinase-B OR matrix-metalloproteinase OR mmp* OR (fibroblast ADJ3 growth-factor-23) OR fgf23 OR fgf-23 OR (somatomedin-binding ADJ3 protein-3) OR (insulin-like ADJ3 growth-factor-binding ADJ3 (protein-3 OR protein-7)) OR igfbp3 OR igfbp-3 OR igfbp7 OR igfbp-7 OR syndecan-1 OR (transcription-factor* ADJ3 (RUNX2 OR RUN-X2 OR RUN-X-2)) OR periostin* OR (transforming ADJ3 (growth-factor*) ADJ3 (beta)) OR tgf-beta OR tgfbeta OR (tissue-inhibitor* ADJ3 metalloproteinase*) OR timp* OR ((procollagen* OR pro-collagen*) ADJ3 (type-I OR type-1 OR type-III OR type-3) ADJ3 (n-terminal OR amino-terminal OR aminoterminal) ADJ3 propeptide*)).ab,ti,kw.) AND (Forecasting / OR Predictive Value of Tests / OR Incidence / OR exp Risk / OR Epidemiology / OR (predict* OR onset* OR incidence* OR risk* OR epidemiolog*).ab,ti,kw.) NOT (news OR congres* OR abstract* OR book* OR chapter*

OR dissertation abstract*).pt. NOT (* heart arrest/ OR * Shock, Cardiogenic / OR * Cardio-Renal Syndrome / OR (acute OR Heart-arrest* OR Sudden-cardiac-death OR Cardiopulmonary-arrest OR cardiac-arrest OR cardi*-shock OR ((cardiorenal OR cardi*-renal) ADJ3 syndrom*) OR emergenc*).ti.) AND (Healthy Volunteers/ OR Population / OR (((healthy OR normal* OR general* OR european* OR american* OR asian* OR african*) ADJ3 (adult* OR human* OR people* OR subject* OR volunteer* OR individual* OR women OR men OR participant*)) OR population* OR communit*).ab,ti,kw. OR adult*.ti.) NOT (exp animals/ NOT humans/) NOT (cell* OR tissue* OR patient*).ti. NOT (Case Reports/ OR (case-report*).ab,ti,kw.) AND English.la.

Embase.com (1971-)

('heart failure'/de OR 'congestive heart failure'/exp OR 'forward heart failure'/de OR 'heart ventricle failure'/exp OR 'systolic dysfunction'/exp OR (((heart OR cardiac OR cardial OR myocard* OR cardis OR ventric*) NEAR/3 (failure* OR insufficien* OR decompensat* OR incompeten* OR dysfunction*))):Ab,ti,kw) AND ('biological marker'/de OR 'cardiac biomarker'/de OR 'natriuretic factor'/de OR 'atrial natriuretic factor'/de OR 'brain natriuretic peptide'/de OR 'amino terminal pro brain natriuretic peptide'/de OR 'cardiac troponin'/de OR troponin/exp OR 'fatty acid binding protein'/exp OR myoglobin/exp OR 'growth differentiation factor'/exp OR myeloperoxidase/de OR ceruloplasmin/de OR 'mid regional proadrenomedullin'/de OR proadrenomedullin/de OR malonaldehyde/de OR 'trypsin inhibitor'/de OR 'alpha 1 antitrypsin'/de OR 'oxidized low density lipoprotein receptor 1'/de OR 'oxidative stress'/de OR 'galectin 3'/de OR 'gelatinase A'/de OR stromelysin/de OR 'neutrophil collagenase'/de OR 'gelatinase B'/de OR 'matrix metalloproteinase'/de OR 'fibroblast growth factor 23'/de OR 'somatomedin binding protein 3'/de OR 'insulin like growth

factor binding protein 7'/de OR 'syndecan 1'/de OR 'transcription factor RUNX2'/de OR 'transforming growth factor beta'/exp OR 'tissue inhibitor of metalloproteinase'/de OR 'tissue inhibitor of metalloproteinase 1'/de OR 'tissue inhibitor of metalloproteinase 2'/de OR 'tissue inhibitor of metalloproteinase 3'/de OR 'tissue inhibitor of metalloproteinase 4'/de OR 'procollagen type i n terminal propeptide'/de OR 'procollagen type 3 aminopropeptide'/de OR ((biological* NEAR/3 marker*) OR biomarker* OR (cardiac* NEAR/3 biological* NEAR/3 marker*) OR ((cardiac* OR cardiovasc*) NEAR/3 (biomarker* OR bio*-marker*)) OR (natriuretic NEAR/3 (factor* OR peptide* OR hormone*)) OR (troponin*) OR probnp OR ntprobnp OR bnp OR tnt OR (fatty-acid NEAR/3 binding NEAR/3 protein*) OR fabp OR myoglobin* OR (growth NEAR/3 differentiat* NEAR/3 factor*) OR gdf OR myeloperoxidas* OR ceruloplasmin* OR proadrenomedullin* OR proadm* OR malonaldehyd* OR malondialdehyd* OR (trypsin NEAR/3 inhibitor*) OR ((alpha-1 OR a-1 OR a1) NEAR/3 protein*) OR (oxidi* NEAR/3 (low-density-lipoprotein OR ldl) NEAR/3 receptor*) OR (oxidative NEAR/3 stress*) OR galectin-3 OR gal-3 OR galectin3 OR gal3 OR gelatinase-A OR stromelysin* OR (neutrophil NEAR/3 collagenas*) OR gelatinase-B OR matrix-metalloproteinase OR mmp* OR (fibroblast NEAR/3 growth-factor-23) OR fgf23 OR fgf-23 OR (somatomedin-binding NEAR/3 protein-3) OR (insulin-like NEAR/3 growth-factor-binding NEAR/3 (protein-3 OR protein-7)) OR igfbp3 OR igfbp-3 OR igfbp7 OR igfbp-7 OR syndecan-1 OR (transcription-factor* NEAR/3 (RUNX2 OR RUN-X2 OR RUN-X-2)) OR periostin* OR (transforming NEAR/3 (growth-factor*) NEAR/3 (β* OR beta)) OR tgf-beta OR tgfbeta OR TGFβ* OR TGF-β* OR (tissue-inhibitor* NEAR/3 metalloproteinase*) OR timp* OR ((procollagen* OR pro-collagen*) NEAR/3 (type-I OR type-1 OR type-III OR type-3) NEAR/3 (n-terminal OR amino-terminal OR aminoterminal) NEAR/3 propeptide*)):Ab,ti,kw) AND ('prediction AND forecasting'/de OR 'prediction'/de OR 'predictive validity'/de OR 'predictive value'/de OR incidence/de OR risk/exp OR

epidemiology/de OR (predict* OR onset* OR incidence* OR risk* OR epidemiolog*):Ab,ti,kw) NOT ([conference abstract]/lim) NOT ('acute heart failure'/mj OR 'heart arrest'/exp/mj OR 'cardiogenic shock'/mj OR 'cardiorenal syndrome'/mj OR (acute OR Heart-arrest* OR Sudden-cardiac-death OR Cardiopulmonary-arrest OR cardiac-arrest OR cardi*-shock OR ((cardiorenal OR cardi*-renal) NEAR/3 syndrom*) OR emergenc*):ti) AND ('normal human'/de OR 'population based case control study'/de OR community/de OR (((healthy OR normal* OR general* OR european* OR american* OR asian* OR african*) NEAR/3 (adult* OR human* OR people* OR subject* OR volunteer* OR individual* OR women OR men OR participant*)) OR population* OR communit*):Ab,ti,kw OR adult*:ti) NOT ([animals]/lim NOT [humans]/lim) NOT (cell* OR tissue* OR patient*):ti NOT ('case report'/de OR (case-report*):Ab,ti,kw) NOT [conference abstract]/lim AND [english]/lim

Web of science Core Collection (1975-)

TS=(((((heart OR cardiac OR cardial OR myocard* OR cardis OR ventric*) NEAR/2 (failure* OR insufficien* OR decompensat* OR incompeten* OR dysfunction*)))) AND (((biological* NEAR/2 marker*) OR biomarker* OR (cardiac* NEAR/2 biological* NEAR/2 marker*) OR ((cardiac* OR cardiovasc*) NEAR/2 (biomarker* OR bio*-marker*)) OR (natriuretic NEAR/2 (factor* OR peptide* OR hormone*)) OR (troponin*) OR probnp OR ntprobnp OR bnp OR tnt OR (fatty-acid NEAR/2 binding NEAR/2 protein*) OR fabp OR myoglobin* OR (growth NEAR/2 differentiat* NEAR/2 factor*) OR gdf OR myeloperoxidas* OR ceruloplasmin* OR proadrenomedullin* OR proadm* OR malonaldehyd* OR malondialdehyd* OR (trypsin NEAR/2 inhibitor*) OR ((alpha-1 OR a-1 OR a1) NEAR/2 protein*) OR (oxidi* NEAR/2 (low-density-lipoprotein OR ldl) NEAR/2 receptor*) OR (oxidative NEAR/2 stress*) OR galectin-3 OR gal-3 OR galectin3 OR gal3 OR gelatinase-A OR stromelysin* OR (neutrophil NEAR/2

collagenas*) OR gelatinase-B OR matrix-metalloproteinase OR mmp* OR (fibroblast NEAR/2 growth-factor-23) OR fgf23 OR fgf-23 OR (somatomedin-binding NEAR/2 protein-3) OR (insulin-like NEAR/2 growth-factor-binding NEAR/2 (protein-3 OR protein-7)) OR igfbp3 OR igfbp-3 OR igfbp7 OR igfbp-7 OR syndecan-1 OR (transcription-factor* NEAR/2 (RUNX2 OR RUN-X2 OR RUN-X-2)) OR periostin* OR (transforming NEAR/2 (growth-factor*) NEAR/2 (beta)) OR tgf-beta OR tgfbeta OR (tissue-inhibitor* NEAR/2 metalloproteinase*) OR timp* OR ((procollagen* OR pro-collagen*) NEAR/2 (type-I OR type-1 OR type-III OR type-3) NEAR/2 (n-terminal OR amino-terminal OR aminoterminal) NEAR/2 propeptide*))) AND ((predict* OR onset* OR incidence* OR risk* OR epidemiolog*)) AND ((((healthy OR normal* OR general* OR european* OR american* OR asian* OR african*) NEAR/2 (adult* OR human* OR people* OR subject* OR volunteer* OR individual* OR women OR men OR participant*)) OR population* OR communit*))) NOT DT=(Meeting Abstract OR Meeting Summary) AND LA=(English) NOT TI=((acute OR Heart-arrest* OR Sudden-cardiac-death OR Cardiopulmonary-arrest OR cardiac-arrest OR cardi*-shock OR ((cardiorenal OR cardi*-renal) NEAR/2 syndrom*) OR emergenc*)) NOT TI=(cell* OR tissue* OR patient*) NOT TI=((case-report*)) AND DT=(article) AND LA=(english)

Cochrane CENTRAL register of trials (1992-)

((((heart OR cardiac OR cardial OR myocard* OR cardis OR ventric*) NEAR/3 (failure* OR insufficien* OR decompensat* OR incompeten* OR dysfunction*))):Ab,ti,kw) AND (((biological* NEAR/3 marker*) OR biomarker* OR (cardiac* NEAR/3 biological* NEAR/3 marker*) OR ((cardiac* OR cardiovasc*) NEAR/3 (biomarker* OR bio* NEXT marker*)) OR (natriuretic NEAR/3 (factor* OR peptide* OR hormone*)) OR (troponin*) OR probnp OR ntprobnp OR bnp OR tnt OR (fatty NEXT acid NEAR/3 binding NEAR/3 protein*) OR fabp OR

myoglobin* OR (growth NEAR/3 differentiat* NEAR/3 factor*) OR gdf OR myeloperoxidas* OR ceruloplasmin* OR proadrenomedullin* OR proadm* OR malonaldehyd* OR malondialdehyd* OR (trypsin NEAR/3 inhibitor*) OR ((alpha NEXT 1 OR a NEXT 1 OR a1) NEAR/3 protein*) OR (oxidi* NEAR/3 (low NEXT density NEXT lipoprotein OR ldl) NEAR/3 receptor*) OR (oxidative NEAR/3 stress*) OR galectin NEXT 3 OR gal NEXT 3 OR galectin3 OR gal3 OR gelatinase NEXT A OR stromelysin* OR (neutrophil NEAR/3 collagenas*) OR gelatinase NEXT B OR matrix NEXT metalloproteinase OR mmp* OR (fibroblast NEAR/3 growth NEXT factor NEXT 23) OR fgf23 OR fgf NEXT 23 OR (somatomedin NEXT binding NEAR/3 protein NEXT 3) OR (insulin NEXT like NEAR/3 growth NEXT factor NEXT binding NEAR/3 (protein NEXT 3 OR protein NEXT 7)) OR igfbp3 OR igfbp NEXT 3 OR igfbp7 OR igfbp NEXT 7 OR syndecan NEXT 1 OR (transcription NEXT factor* NEAR/3 (RUNX2 OR RUN NEXT X2 OR RUN NEXT X NEXT 2)) OR periostin* OR (transforming NEAR/3 (growth NEXT factor*) NEAR/3 (β* OR beta)) OR tgf NEXT beta OR tgfbeta OR TGFβ* OR TGF NEXT β* OR (tissue NEXT inhibitor* NEAR/3 metalloproteinase*) OR timp* OR ((procollagen* OR pro NEXT collagen*) NEAR/3 (type NEXT I OR type NEXT 1 OR type NEXT III OR type NEXT 3) NEAR/3 (n NEXT terminal OR amino NEXT terminal OR aminoterminal) NEAR/3 propeptide*)):Ab,ti,kw) AND ((predict* OR onset* OR incidence* OR risk* OR epidemiolog*):Ab,ti,kw) AND ((((healthy OR normal* OR general* OR european* OR american* OR asian* OR african*) NEAR/3 (adult* OR human* OR people* OR subject* OR volunteer* OR individual* OR women OR men OR participant*)) OR population* OR communit*):Ab,ti,kw)

Additional Search

Medline ALL OVID (1946-)

(exp Heart Failure / OR (((heart OR cardiac OR cardial OR myocard* OR cardis OR ventric*) ADJ3 (failure* OR insufficien* OR decompensat* OR incompeten* OR

dysfunction*))).ab,ti,kw.) AND (exp Biomarkers/ OR exp * Natriuretic Agents/ OR * Atrial Natriuretic Factor/ OR exp * Natriuretic Peptide, Brain/ OR exp * Troponin/ OR exp * Fatty Acid-Binding Proteins/ OR * Myoglobin/ OR exp * Growth Differentiation Factors/ OR * Peroxidase/ OR * Ceruloplasmin/ OR * Adrenomedullin / OR * Malondialdehyde/ OR exp * Trypsin Inhibitors/ OR * alpha 1-Antitrypsin/ OR * Scavenger Receptors, Class E/ OR * Oxidative Stress/ OR * Galectin 3/ OR exp * Matrix Metalloproteinases/ OR * Fibroblast Growth Factor-23/ OR exp * Insulin-Like Growth Factor Binding Proteins/ OR * Syndecan-1/ OR * Transforming Growth Factor beta / OR exp * Tissue Inhibitor of Metalloproteinases/ OR ((biological* ADJ3 marker*) OR biomarker* OR (cardiac* ADJ3 biological* ADJ3 marker*) OR ((cardiac* OR cardiovasc*) ADJ3 (biomarker* OR bio*-marker*)) OR (natriuretic ADJ3 (factor* OR peptide* OR hormone*)) OR (troponin*) OR probnp OR ntprobnp OR bnp OR tnt OR (fatty-acid ADJ3 binding ADJ3 protein*) OR fabp OR myoglobin* OR (growth ADJ3 differentiat* ADJ3 factor*) OR gdf OR myeloperoxidas* OR ceruloplasmin* OR proadrenomedullin* OR proadm* OR malonaldehyd* OR malondialdehyd* OR (trypsin ADJ3 inhibitor*) OR ((alpha-1 OR a-1 OR a1) ADJ3 protein*) OR (oxidi* ADJ3 (low-density-lipoprotein OR ldl) ADJ3 receptor*) OR (oxidative ADJ3 stress*) OR galectin-3 OR gal-3 OR galectin3 OR gal3 OR gelatinase-A OR stromelysin* OR (neutrophil ADJ3 collagenas*) OR gelatinase-B OR matrix-metalloproteinase OR mmp* OR (fibroblast ADJ3 growth-factor-23) OR fgf23 OR fgf-23 OR (somatomedin-binding ADJ3 protein-3) OR (insulin-like ADJ3 growth-factor-binding ADJ3 (protein-3 OR protein-7)) OR igfbp3 OR igfbp-3 OR igfbp7 OR igfbp-7 OR syndecan-1 OR (transcription-factor* ADJ3 (RUNX2 OR RUN-X2 OR RUN-X-2)) OR periostin* OR (transforming ADJ3 (growth-factor*) ADJ3 (beta)) OR tgf-beta OR tgfbeta OR (tissue-inhibitor* ADJ3 metalloproteinase*) OR timp* OR ((procollagen* OR pro-collagen*) ADJ3 (type-I OR type-1 OR type-III OR type-3) ADJ3 (n-terminal OR amino-terminal OR aminoterminal) ADJ3 propeptide*)).ti.) AND (* Forecasting /

OR * Predictive Value of Tests / OR * Incidence / OR exp * Risk / OR * Epidemiology / OR (predict* OR onset* OR incidence* OR risk* OR epidemiolog*).ti.) NOT (news OR congres* OR abstract* OR book* OR chapter* OR dissertation abstract*).pt. NOT (* heart arrest/ OR * Shock, Cardiogenic / OR * Cardio-Renal Syndrome / OR (acute OR Heart-arrest* OR Sudden-cardiac-death OR Cardiopulmonary-arrest OR cardiac-arrest OR cardi*-shock OR ((cardiorenal OR cardi*-renal) ADJ3 syndrom*) OR emergenc*).ti.) NOT (Healthy Volunteers/ OR Population / OR (((healthy OR normal* OR general* OR european* OR american* OR asian* OR african*) ADJ3 (adult* OR human* OR people* OR subject* OR volunteer* OR individual* OR women OR men OR participant*)) OR population* OR communit*).ab,ti,kw. OR adult*.ti.) NOT (exp animals/ NOT humans/) NOT (cell* OR tissue* OR patient*).ti. NOT (Case Reports/ OR (case-report*).ab,ti,kw.)

Embase.com (1971-)

('heart failure'/de OR 'congestive heart failure'/exp OR 'forward heart failure'/de OR 'heart ventricle failure'/exp OR 'systolic dysfunction'/exp OR (((heart OR cardiac OR cardial OR myocard* OR cardis OR ventric*) NEAR/3 (failure* OR insufficien* OR decompensat* OR incompeten* OR dysfunction*))):ab,ti,kw) AND ('biological marker'/mj OR 'cardiac biomarker'/mj OR 'natriuretic factor'/mj OR 'atrial natriuretic factor'/mj OR 'brain natriuretic peptide'/mj OR 'amino terminal pro brain natriuretic peptide'/mj OR 'cardiac troponin'/mj OR troponin/mj/exp OR 'fatty acid binding protein'/mj/exp OR myoglobin/mj/exp OR 'growth differentiation factor'/mj/exp OR myeloperoxidase/mj OR ceruloplasmin/mj OR 'mid regional proadrenomedullin'/mj OR proadrenomedullin/mj OR malonaldehyde/mj OR 'trypsin inhibitor'/mj OR 'alpha 1 antitrypsin'/mj OR 'oxidized low density lipoprotein receptor 1'/mj OR 'oxidative stress'/mj OR 'galectin 3'/mj OR 'gelatinase A'/mj OR stromelysin/mj OR

'neutrophil collagenase'/mj OR 'gelatinase B'/mj OR 'matrix metalloproteinase'/mj OR 'fibroblast growth factor 23'/mj OR 'somatomedin binding protein 3'/mj OR 'insulin like growth factor binding protein 7'/mj OR 'syndecan 1'/mj OR 'transcription factor RUNX2'/mj OR 'transforming growth factor beta'/mj/exp OR 'tissue inhibitor of metalloproteinase'/mj OR 'tissue inhibitor of metalloproteinase 1'/mj OR 'tissue inhibitor of metalloproteinase 2'/mj OR 'tissue inhibitor of metalloproteinase 3'/mj OR 'tissue inhibitor of metalloproteinase 4'/mj OR 'procollagen type i n terminal propeptide'/mj OR 'procollagen type 3 aminopropeptide'/mj OR ((biological* NEAR/3 marker*) OR biomarker* OR (cardiac* NEAR/3 biological* NEAR/3 marker*) OR ((cardiac* OR cardiovasc*) NEAR/3 (biomarker* OR bio*-marker*)) OR (natriuretic NEAR/3 (factor* OR peptide* OR hormone*)) OR (troponin*) OR probnp OR ntprobnp OR bnp OR tnt OR (fatty-acid NEAR/3 binding NEAR/3 protein*) OR fabp OR myoglobin* OR (growth NEAR/3 differentiat* NEAR/3 factor*) OR gdf OR myeloperoxidas* OR ceruloplasmin* OR proadrenomedullin* OR proadm* OR malonaldehyd* OR malondialdehyd* OR (trypsin NEAR/3 inhibitor*) OR ((alpha-1 OR a-1 OR a1) NEAR/3 protein*) OR (oxidi* NEAR/3 (low-density-lipoprotein OR ldl) NEAR/3 receptor*) OR (oxidative NEAR/3 stress*) OR galectin-3 OR gal-3 OR galectin3 OR gal3 OR gelatinase-A OR stromelysin* OR (neutrophil NEAR/3 collagenas*) OR gelatinase-B OR matrix-metalloproteinase OR mmp* OR (fibroblast NEAR/3 growth-factor-23) OR fgf23 OR fgf-23 OR (somatomedin-binding NEAR/3 protein-3) OR (insulin-like NEAR/3 growth-factor-binding NEAR/3 (protein-3 OR protein-7)) OR igfbp3 OR igfbp-3 OR igfbp7 OR igfbp-7 OR syndecan-1 OR (transcription-factor* NEAR/3 (RUNX2 OR RUN-X2 OR RUN-X-2)) OR periostin* OR (transforming NEAR/3 (growth-factor*) NEAR/3 (β* OR beta)) OR tgf-beta OR tgfbeta OR TGFβ* OR TGF-β* OR (tissue-inhibitor* NEAR/3 metalloproteinase*) OR timp* OR ((procollagen* OR pro-collagen*) NEAR/3 (type-I OR type-1 OR type-III OR type-3) NEAR/3 (n-terminal OR amino-terminal OR aminoterminal)

NEAR/3 propeptide*)):ti) AND ('prediction AND forecasting'/mj/de OR 'prediction'/mj/de OR 'predictive validity'/mj/de OR 'predictive value'/mj/de OR incidence/mj/de OR risk/mj/exp OR epidemiology/mj/de OR (predict* OR onset* OR incidence* OR risk* OR epidemiolog*):ti) NOT ([conference abstract]/lim) NOT ('acute heart failure'/mj OR 'heart arrest'/exp/mj OR 'cardiogenic shock'/mj OR 'cardiorenal syndrome'/mj OR (acute OR Heart-arrest* OR Sudden-cardiac-death OR Cardiopulmonary-arrest OR cardiac-arrest OR cardi*-shock OR ((cardiorenal OR cardi*-renal) NEAR/3 syndrom*) OR emergenc*):ti) NOT ('normal human'/de OR 'population based case control study'/de OR community/de OR (((healthy OR normal* OR general* OR european* OR american* OR asian* OR african*) NEAR/3 (adult* OR human* OR people* OR subject* OR volunteer* OR individual* OR women OR men OR participant*)) OR population* OR communit*):Ab,ti,kw OR adult*:ti) NOT ([animals]/lim NOT [humans]/lim) NOT (cell* OR tissue* OR patient*):ti NOT ('case report'/de OR (case-report*):Ab,ti,kw)

Web of science Core Collection (1975-)

TS=((((heart OR cardiac OR cardial OR myocard* OR cardis OR ventric*) NEAR/2 (failure* OR insufficien* OR decompensat* OR incompeten* OR dysfunction*)))) AND TI=(((biological* NEAR/2 marker*) OR biomarker* OR (cardiac* NEAR/2 biological* NEAR/2 marker*) OR ((cardiac* OR cardiovasc*) NEAR/2 (biomarker* OR bio*-marker*)) OR (natriuretic NEAR/2 (factor* OR peptide* OR hormone*)) OR (troponin*) OR probnp OR ntprobnp OR bnp OR tnt OR (fatty-acid NEAR/2 binding NEAR/2 protein*) OR fabp OR myoglobin* OR (growth NEAR/2 differentiat* NEAR/2 factor*) OR gdf OR myeloperoxidas* OR ceruloplasmin* OR proadrenomedullin* OR proadm* OR malonaldehyd* OR malondialdehyd* OR (trypsin NEAR/2 inhibitor*) OR ((alpha-1 OR a-1 OR a1) NEAR/2 protein*) OR (oxidi* NEAR/2 (low-density-lipoprotein OR ldl) NEAR/2 receptor*) OR (oxidative NEAR/2 stress*) OR galectin-3 OR

gal-3 OR galectin3 OR gal3 OR gelatinase-A OR stromelysin* OR (neutrophil NEAR/2 collagenas*) OR gelatinase-B OR matrix-metalloproteinase OR mmp* OR (fibroblast NEAR/2 growth-factor-23) OR fgf23 OR fgf-23 OR (somatomedin-binding NEAR/2 protein-3) OR (insulin-like NEAR/2 growth-factor-binding NEAR/2 (protein-3 OR protein-7)) OR igfbp3 OR igfbp-3 OR igfbp7 OR igfbp-7 OR syndecan-1 OR (transcription-factor* NEAR/2 (RUNX2 OR RUN-X2 OR RUN-X-2)) OR periostin* OR (transforming NEAR/2 (growth-factor*) NEAR/2 (beta)) OR tgf-beta OR tgfbeta OR (tissue-inhibitor* NEAR/2 metalloproteinase*) OR timp* OR ((procollagen* OR pro-collagen*) NEAR/2 (type-I OR type-1 OR type-III OR type-3) NEAR/2 (n-terminal OR amino-terminal OR aminoterminal) NEAR/2 propeptide*))) AND TI=((predict* OR onset* OR incidence* OR risk* OR epidemiolog*)) NOT TS=((((healthy OR normal* OR general* OR european* OR american* OR asian* OR african*) NEAR/2 (adult* OR human* OR people* OR subject* OR volunteer* OR individual* OR women OR men OR participant*)) OR population* OR communit*)) NOT DT=(Meeting Abstract OR Meeting Summary) AND LA=(English) NOT TI=((acute OR Heart-arrest* OR Sudden-cardiac-death OR Cardiopulmonary-arrest OR cardiac-arrest OR cardi*-shock OR ((cardiorenal OR cardi*-renal) NEAR/2 syndrom*) OR emergenc*)) NOT TI=(cell* OR tissue* OR patient*) NOT TI=((case-report*))

Cochrane CENTRAL register of trials (1992-)

((((heart OR cardiac OR cardial OR myocard* OR cardis OR ventric*) NEAR/3 (failure* OR insufficien* OR decompensat* OR incompeten* OR dysfunction*))):ab,ti,kw) AND (((biological* NEAR/3 marker*) OR biomarker* OR (cardiac* NEAR/3 biological* NEAR/3 marker*) OR ((cardiac* OR cardiovasc*) NEAR/3 (biomarker* OR bio* NEXT marker*)) OR (natriuretic NEAR/3 (factor* OR peptide* OR hormone*)) OR (troponin*) OR probnp OR ntprobnp OR bnp OR tnt OR (fatty NEXT acid NEAR/3 binding NEAR/3 protein*) OR fabp OR

myoglobin* OR (growth NEAR/3 differentiat* NEAR/3 factor*) OR gdf OR myeloperoxidas* OR ceruloplasmin* OR proadrenomedullin* OR proadm* OR malonaldehyd* OR malondialdehyd* OR (trypsin NEAR/3 inhibitor*) OR ((alpha NEXT 1 OR a NEXT 1 OR a1) NEAR/3 protein*) OR (oxidi* NEAR/3 (low NEXT density NEXT lipoprotein OR ldl) NEAR/3 receptor*) OR (oxidative NEAR/3 stress*) OR galectin NEXT 3 OR gal NEXT 3 OR galectin3 OR gal3 OR gelatinase NEXT A OR stromelysin* OR (neutrophil NEAR/3 collagenas*) OR gelatinase NEXT B OR matrix NEXT metalloproteinase OR mmp* OR (fibroblast NEAR/3 growth NEXT factor NEXT 23) OR fgf23 OR fgf NEXT 23 OR (somatomedin NEXT binding NEAR/3 protein NEXT 3) OR (insulin NEXT like NEAR/3 growth NEXT factor NEXT binding NEAR/3 (protein NEXT 3 OR protein NEXT 7)) OR igfbp3 OR igfbp NEXT 3 OR igfbp7 OR igfbp NEXT 7 OR syndecan NEXT 1 OR (transcription NEXT factor* NEAR/3 (RUNX2 OR RUN NEXT X2 OR RUN NEXT X NEXT 2)) OR periostin* OR (transforming NEAR/3 (growth NEXT factor*) NEAR/3 (β* OR beta)) OR tgf NEXT beta OR tgfbeta OR TGFβ* OR TGF NEXT β* OR (tissue NEXT inhibitor* NEAR/3 metalloproteinase*) OR timp* OR ((procollagen* OR pro NEXT collagen*) NEAR/3 (type NEXT I OR type NEXT 1 OR type NEXT III OR type NEXT 3) NEAR/3 (n NEXT terminal OR amino NEXT terminal OR aminoterminal) NEAR/3 propeptide*)):Ti) AND ((predict* OR onset* OR incidence* OR risk* OR epidemiolog*):Ti) NOT ((((healthy OR normal* OR general* OR european* OR american* OR asian* OR african*) NEAR/3 (adult* OR human* OR people* OR subject* OR volunteer* OR individual* OR women OR men OR participant*)) OR population* OR communit*):ab,ti,kw)

Supplemental Figures

Author	Total N	HF events N	HR
**NT-proBNP**			
Yan et al, 2020	48,455	1,990 (4.1%)	1.72
Nambi et al, 2013	9,868	970 (9.8%)	1.75
Brouwers et al, 2014	8,569	374 (4.4%)	2.12
Funke-Kaiser et al, 2014	7,899	505 (6.4%)	1.72
Segar et al, 2021	5,210	232 (4.5%)	2.77
Smith et al, 2010	5,135	112 (2.2%)	1.95
Harris et al, 2010	4,312	710 (16.5%)	1.52
Wu et al, 2022 (ViDA)	4,102	248 (6.0%)	2.90
Wannamethee et al, 2014	3,672	257 (7.0%)	2.15
Ho et al, 2018	3,523	226 (6.4%)	1.79
Wu et al, 2022 (HVOLS)	2,528	227 (9.0%)	2.25
Lind et al, 2022	2,449	132 (5.4%)	2.62
Frary et al, 2020	1,951	-	2.33
AbouEzzeddine et al, 2016	1,824	173 (9.5%)	2.00
**Pooled**	**109,497**	**6,156 (5.6%)**	**2.03**
**hs-cTnI**			
Yan et al, 2020	48,455	1,990 (4.1%)	1.42
Welsh et al, 2019	19,285	216 (1.1%)	1.86
Neumann et al, 2014	7,899	505 (6.4%)	1.19
Tang et al, 2021	5,876	206 (3.5%)	1.58
Wu et al, 2022 (ViDA)	4,102	248 (6.0%)	1.85
Wang et al, 2012	3,428	162 (4.7%)	1.28
Zhu et al, 2018 (1)	3,215	121 (3.8%)	1.43
Wu et al, 2022 (HVOLS)	2,528	227 (9.0%)	2.10
Saha et al, 2022	2,423	139 (5.7%)	2.13
McKie et al, 2014	1,843	193 (10.5%)	1.62
AbouEzzeddine et al, 2016	1,824	173 (9.5%)	1.66
**Pooled**	**100,878**	**4,180 (4.1%)**	**1.58**
**hs-cTnT**			
Welsh et al, 2019 (1)	19,285	216 (1.1%)	1.69
Nambi et al, 2013	9,868	970 (9.8%)	1.10
Brouwers et al, 2014	8,569	374 (4.4%)	1.56
Nguyen et al, 2020	5,584	262 (4.7%)	1.89
Wu et al, 2022 (ViDA)	4,102	248 (1.2%)	3.91
Welsh et al, 2019 (2)	3,852	295 (7.7%)	1.58
Wu et al, 2022 (HVOLS)	2,528	227 (1.4%)	2.20
**Pooled**	**53,788**	**2,592 (4.8%)**	**1.74**
**MR-proANP**			
Brouwers et al, 2014	8,569	374 (4.4%)	1.62
Funke-Kaiser et al, 2014	7,899	505 (6.4%)	1.45
Smith et al, 2010	5,135	112 (2.2%)	1.68
**Pooled**	**21,603**	**991 (4.6%)**	**1.54**
**BNP**			
Funke-Kaiser et al, 2014	7,899	505 (6.4%)	1.59
Velageleti et al, 2010	2,754	95 (3.4%)	1.52
AbouEzzeddine et al, 2016	1,824	173 (9.5%)	1.56
**Pooled**	**12,477**	**773 (6.2%)**	**1.57**
**FABP4**			
Djousse et al, 2013	4,260	1,182 (27.7%)	1.09
Molvin et al, 2020	1,713	130 (7.6%)	0.95
Stenemo et al, 2018	1,586	170 (8.9%)	0.99
**Pooled**	**7,559**	**1,482 (19.6%)**	**1.06**

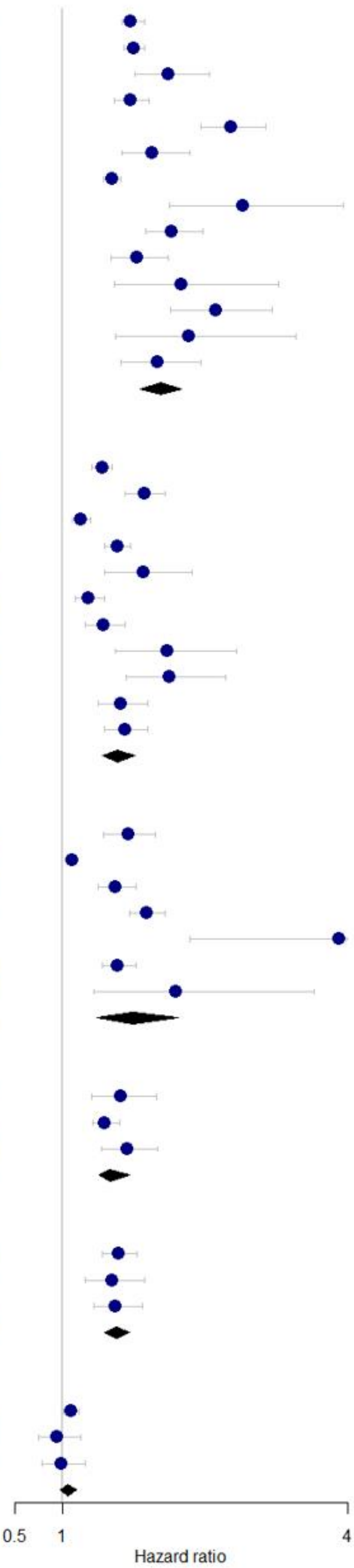

Figure A.1. Cardiac Biomarkers and Incident HF. Forest plots depicting associations between NT-proBNP, hs-cTnI, hs-cTnT, MR-proANP, BNP, and FABP4 with incident HF. Data from 26 studies (180,860 observations) were pooled using random-effects models. Heterogeneity (I^2): NT-proBNP 89%, hs-cTnI 86%, hs-cTnT 97%, MR-proANP 21%, BNP 0%, FABP4 0%. *BNP, b-type natriuretic peptide; FABP4, fatty-acid binding protein 4; HF, heart failure; HR, hazard ratio; hs-cTnI, high-sensitivity cardiac troponin I; hs-cTnT, high-sensitivity cardiac troponin T; MR-proANP, midregional pro-atrial natriuretic peptide; N, number; NT-proBNP, N-terminal pro b-type natriuretic peptide.*

Author	Total N	HF events N	HR
**hs-cTnI - Sensitivity analysis**			
Yan et al, 2020	48,455	1,990 (4.1%)	1.42
Welsh et al, 2019	19,285	216 (1.1%)	1.86
Tang et al, 2021	5,876	206 (3.5%)	1.58
Wu et al, 2022 (ViDA)	4,102	248 (6.0%)	1.85
Zhu et al, 2018	3,215	121 (3.8%)	1.43
Wu et al, 2022 (HVOLS)	2,528	227 (9.0%)	2.10
Saha et al, 2022	2,423	139 (5.7%)	2.13
McKie et al, 2014	1,843	193 (10.5%)	1.62
AbouEzzeddine et al, 2016	1,824	173 (9.5%)	1.66
**Pooled**	**100,878**	**4,180 (4.1%)**	**1.58**
**hs-cTnT - Log-transformed**			
Welsh et al, 2019 (1)	19,285	216 (1.1%)	1.69
Nguyen et al, 2020	5,584	262 (4.7%)	1.89
Wu et al, 2022 (ViDA)	4,102	248 (1.2%)	3.91
Welsh et al, 2019 (2)	3,852	295 (7.7%)	1.58
Wu et al, 2022 (HVOLS)	2,528	227 (1.4%)	2.20
**Pooled**	**35,351**	**1,248 (3.5%)**	**1.95**
**hs-cTnT - Sensitivity analysis**			
Welsh et al, 2019 (1)	19,285	216 (1.1%)	1.69
Brouwers et al, 2014	8,569	374 (4.4%)	1.56
Nguyen et al, 2020	5,584	262 (4.7%)	1.89
Welsh et al, 2019 (2)	3,852	295 (7.7%)	1.58
Wu et al, 2022 (HVOLS)	2,528	227 (1.4%)	2.20
**Pooled**	**39,818**	**1,374 (3.5%)**	**1.70**
**FABP4 - Simple multivariate**			
Djousse et al, 2013	4,260	1,182 (27.7%)	1.32
Molvin et al, 2020	1,713	130 (7.6%)	1.38
Stenemo et al, 2018 (PIVUS)	901	80 (8.9%)	1.72
Stenemo et al, 2018 (ULSAM)	685	90 (13.1%)	1.33
**Pooled**	**7,559**	**1,482 (19.6%)**	**1.34**

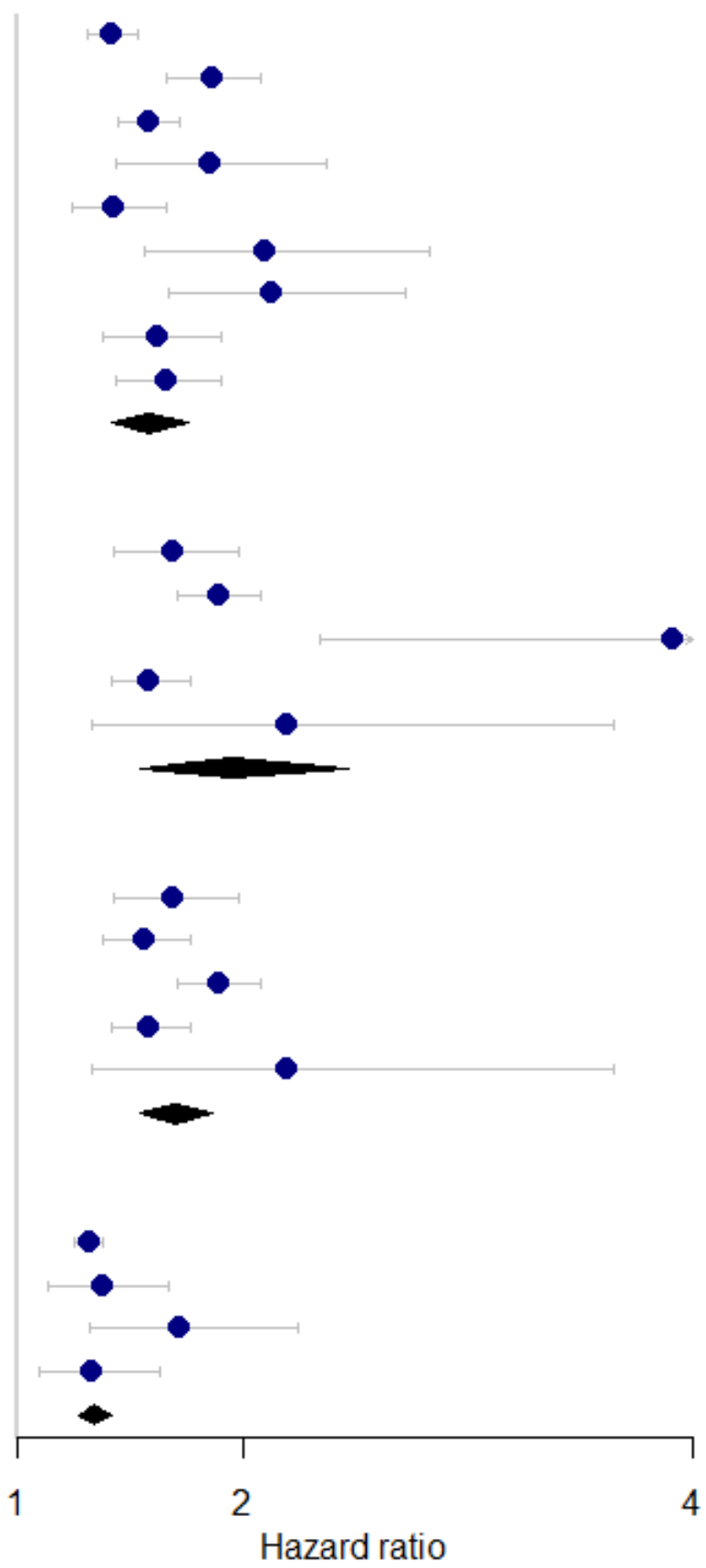

Figure A.2. Sensitivity Analysis of Cardiac Biomarkers. Additional Forest Plots Showing the Association Between Cardiac Biomarkers (hs-cTnI, hs-cTnT, FABP4) and Incident Heart Failure. The I^2 were 0% for simple multivariate models of hs-cTnI, 69% for log-transformed HRs of hs-cTnT, and 55% for sensitivity analysis for heterogeneity among the studies. *FABP4, fatty-acid binding protein 4; HF, heart failure; HR, hazard ratio; hs-cTnI, high-sensitivity cardiac troponin I; hs-cTnT, high-sensitivity cardiac troponin T; N, number.*

Author	Total N	HF events N	HR
**GDF-15**			
Echouffo-Tcheugui et al, 2022	10,570	2,429 (23.0%)	1.64
Fluschnik et al, 2018	3,785	-	1.36
Ho et al, 2018	3,523	226 (6.4%)	1.78
Wang et al, 2012	3,428	162 (4.7%)	1.52
Stenemo et al, 2018	1,586	170 (10.7%)	1.30
**Pooled**	**22,892**	**2,987 (13.0%)**	**1.51**
**MR-proADM**			
Brouwers et al, 2014	8,569	374 (4.4%)	1.10
Funke-Kaiser et al, 2014	7,899	505 (6.4%)	1.67
Smith et al, 2010	5,135	112 (2.2%)	1.35
**Pooled**	**21,603**	**991 (4.6%)**	**1.38**
**MPO**			
Tang et al, 2009	3,733	569 (15.2%)	1.34
Ho et al, 2018	3,523	226 (6.4%)	1.30
Oyenuga et al, 2018	1,465	163 (11.1%)	0.96
**Pooled**	**8,721**	**958 (11.0%)**	**1.17**

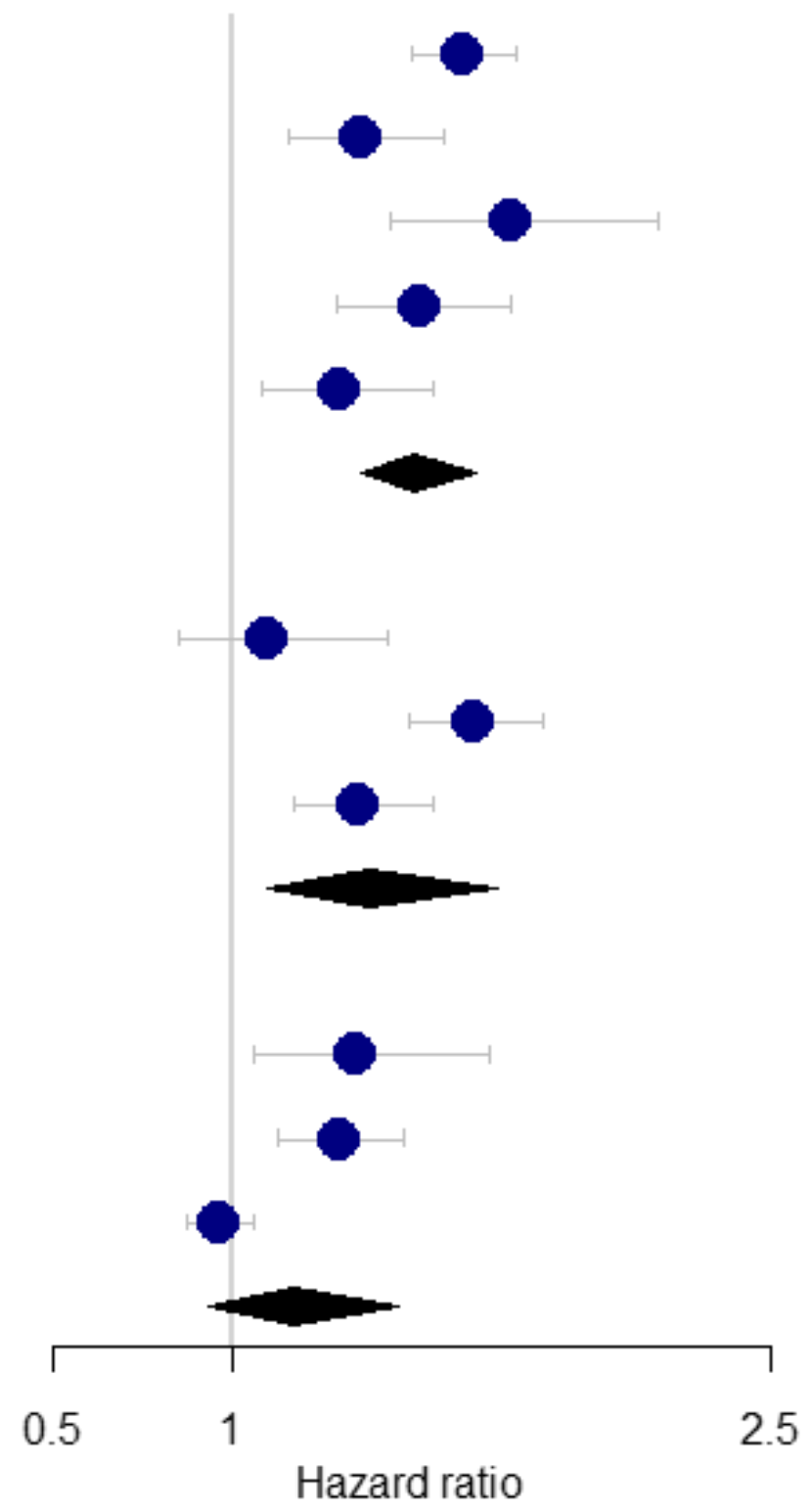

Figure A.3. Oxidative Stress Biomarkers and Incident HF. Forest plots depicting associations between GDF-15, MR-proADM, and MPO with incident HF. Data from 10 studies (49,693 observations) were pooled using random-effects models. Heterogeneity (I^2): GDF-15 58%, MR-proADM 82%, MPO 87%. *GDF-15, growth differentiation factor 15; HF, heart failure; HR, hazard ratio; MPO, myeloperoxidase; MR-proADM, midregional proadrenomedullin; N, number.*

Author	Total N	HF events N	HR
**FGF-23**			
Lutsey et al, 2014	11,638	1,515 (13.0%)	1.11
Binnenmars et al, 2022	6,830	227 (3.3%)	1.29
Almahmoud et al, 2018	6,542	291 (4.4%)	1.17
Ho et al, 2018	3,523	226 (6.4%)	1.20
Paul et al, 2021	3,152	33 (1.0%)	1.52
Stenemo et al, 2018	1,586	170 (10,7%)	1.16
Giuseppe et al, 2014	1,349	221 (16.4%)	1.29
Sharma et al, 2020	844	220 (26.1%)	0.97
**Pooled**	**34,779**	**2,813 (8.1%)**	**1.17**
**Galectin-3**			
Brouwers et al, 2014	8,569	374 (4.4%)	1.10
Jagodzinski et al, 2015	8,312	641 (7.7%)	1.10
Aguilar et al, 2020	4,829	217 (4.5%)	3.90
Ho et al, 2012	3,353	166 (5.0%)	1.27
Ghorbani et al, 2018	2,477	108 (4.4%)	1.39
AbouEzzeddine et al, 2016	1,824	173 (9.5%)	1.17
McEvoy et al, 2016	1,809	167 (9.2%)	1.47
Stenemo et al, 2018 (PIVUS)	901	80 (8.9%)	1.21
Stenemo et al, 2018 (ULSAM)	685	90 (13.1%)	1.19
**Pooled**	**32,759**	**2,016 (6.2%)**	**1.23**
**PAI-1**			
Suthahar et al, 2021	8,202	357 (4.4%)	1.00
Ho et al, 2018	3,523	226 (6.4%)	1.10
Molvin et al, 2020	1,713	130 (7.6%)	1.00
**Pooled**	**13,438**	**713 (5.3%)**	**1.04**

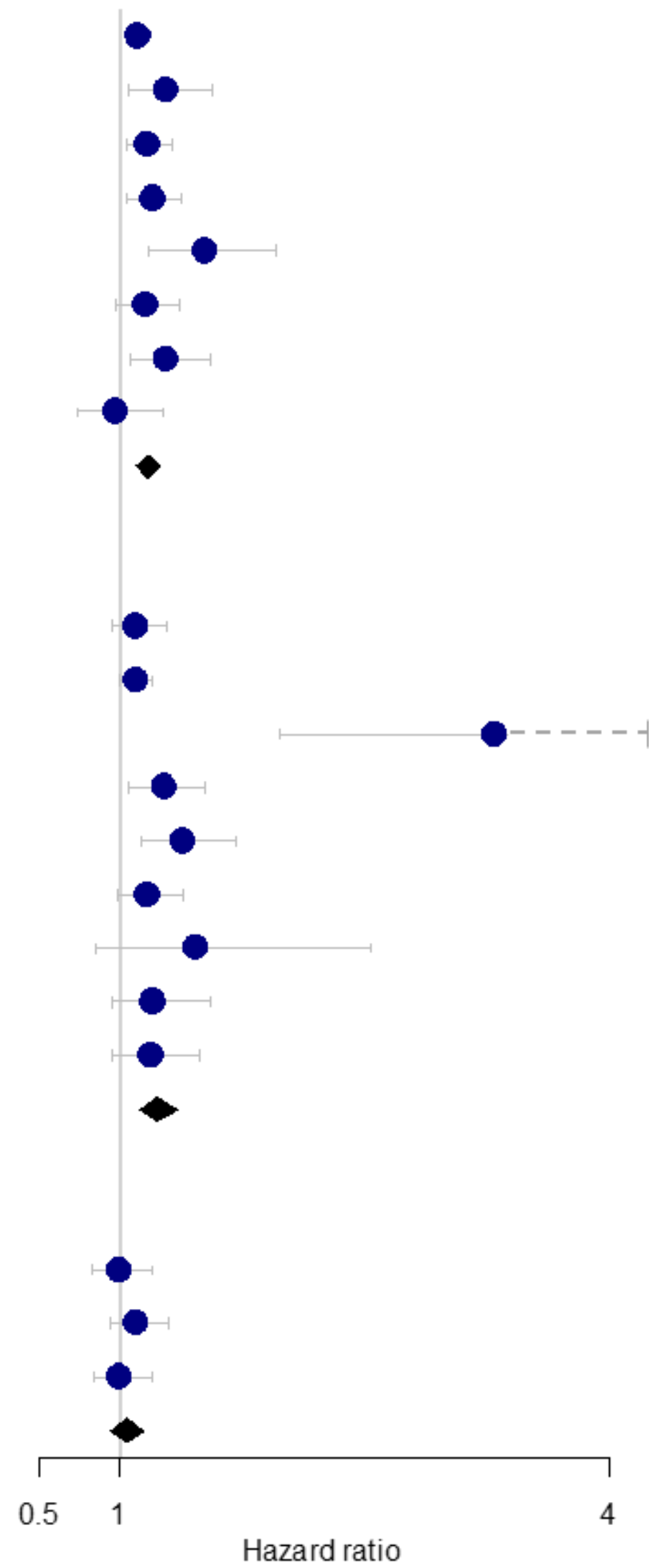

Figure A.4. Fibrosis Biomarkers and Incident HF. Forest plot depicting associations between FGF-23, Gal-3, and PAI-1 with incident HF. Data from 17 studies (76,552 observations) were pooled using random-effects models. Heterogeneity (I^2): FGF-23 43%, Gal-3 64%, PAI-1 0%.

FGF-23, fibroblast growth factor 23; Gal-3, galectin-3; HF, heart failure; HR, hazard ratio; N, number; PAI-1, plasminogen activator inhibitor-1.

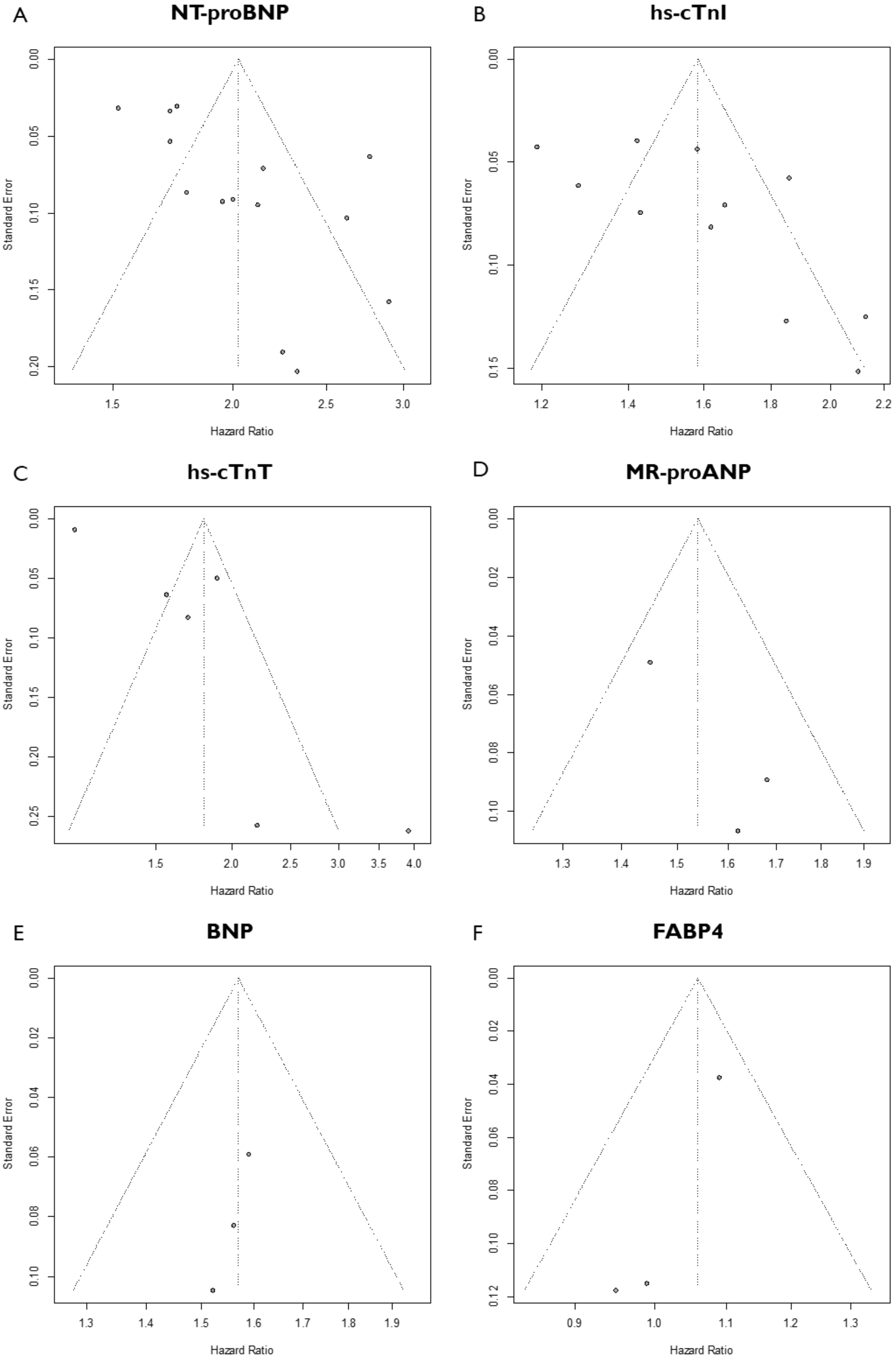

A
NT-proBNP
Standard Error
0.00
0.05
0.10
0.15
0.20
1.5
2.0
2.5
3.0
Hazard Ratio
B
hs-cTnI
Standard Error
0.00
0.05
0.10
0.15
1.2
1.4
1.6
1.8
2.0
2.2
Hazard Ratio
C
hs-cTnT
Standard Error
0.00
0.05
0.10
0.15
0.20
0.25
1.5
2.0
2.5
3.0
3.5
4.0
Hazard Ratio
D
MR-proANP
Standard Error
0.00
0.02
0.04
0.06
0.08
0.10
1.3
1.4
1.5
1.6
1.7
1.8
1.9
Hazard Ratio
E
BNP
Standard Error
0.00
0.02
0.04
0.06
0.08
0.10
1.3
1.4
1.5
1.6
1.7
1.8
1.9
Hazard Ratio
F
FABP4
Standard Error
0.00
0.02
0.04
0.06
0.08
0.10
0.12
0.9
1.0
1.1
1.2
1.3
Hazard Ratio

Figure A.5. Funnel Plots Evaluating Publication Bias in Cardiac Biomarker Studies. *BNP, b-type natriuretic peptide; FABP4, fatty acid binding protein 4; hs-cTnI, high-sensitivity cardiac troponin I; hs-cTnT, high-sensitivity cardiac troponin T; MR-proANP, midregional pro-atrial natriuretic peptide; NT-proBNP, N-terminal pro b-type natriuretic peptide.*

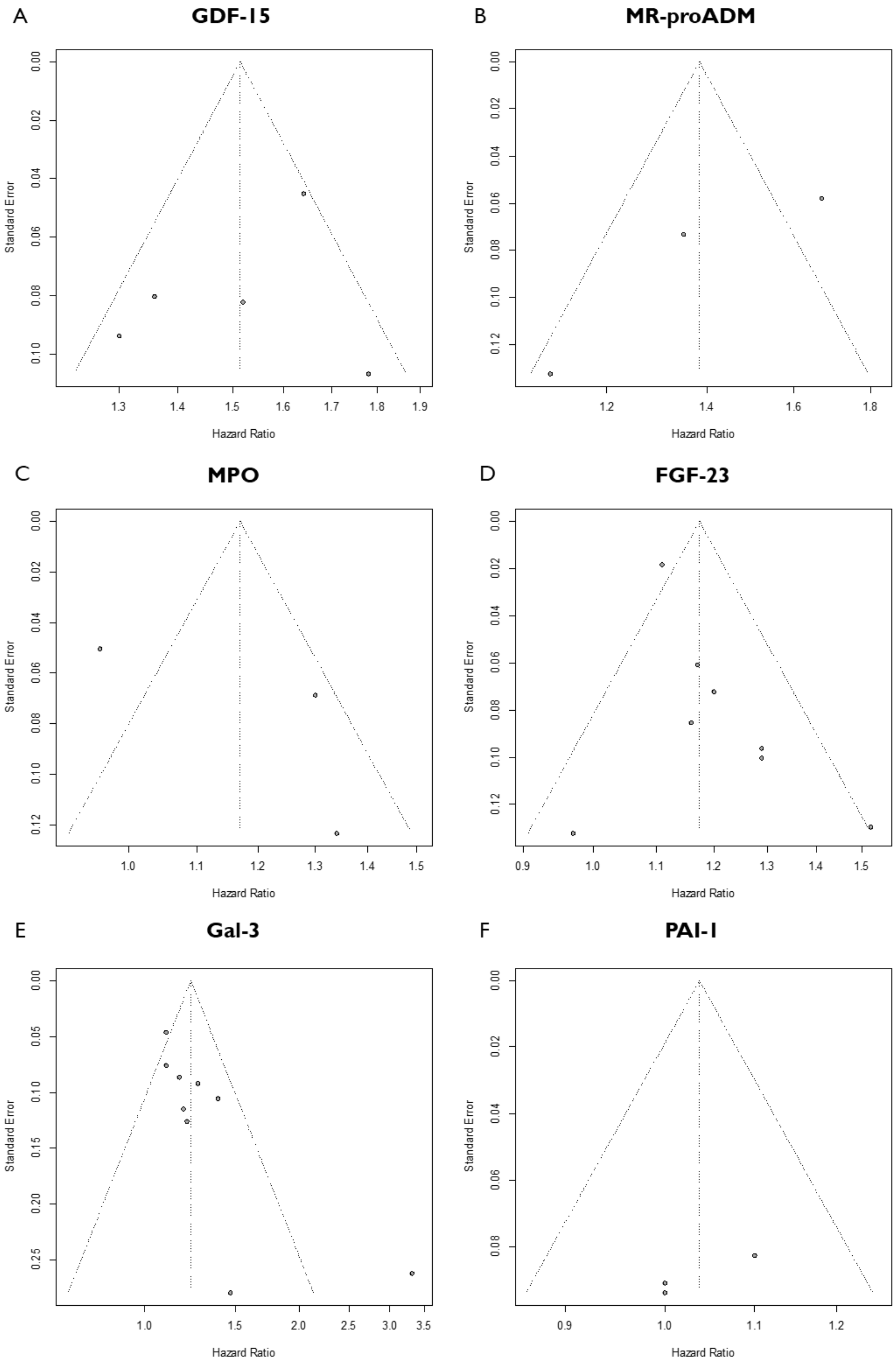
A
GDF-15
Standard Error
0.00
0.02
0.04
0.06
0.08
0.10
1.3
1.4
1.5
1.6
1.7
1.8
1.9
Hazard Ratio
B
MR-proADM
Standard Error
0.00
0.02
0.04
0.06
0.08
0.10
0.12
1.2
1.4
1.6
1.8
Hazard Ratio
C
MPO
Standard Error
0.00
0.02
0.04
0.06
0.08
0.10
0.12
1.0
1.1
1.2
1.3
1.4
1.5
Hazard Ratio
D
FGF-23
Standard Error
0.00
0.02
0.04
0.06
0.08
0.10
0.12
0.9
1.0
1.1
1.2
1.3
1.4
1.5
Hazard Ratio
E
Gal-3
Standard Error
0.00
0.05
0.10
0.15
0.20
0.25
1.0
1.5
2.0
2.5
3.0
3.5
Hazard Ratio
F
PAI-1
Standard Error
0.00
0.02
0.04
0.06
0.08
0.9
1.0
1.1
1.2
Hazard Ratio

Figure A.6. Funnel Plots Evaluating Publication Bias in Oxidative Stress and Fibrosis-Related Biomarker Studies. *FGF-23, fibroblast growth factor 23; GDF-15, growth differentiation factor 15; MPO, myeloperoxidase; MR-proADM, midregional proadrenomedullin; PAI-1, plasminogen activator inhibitor-1.*

Supplemental Tables

Table A.1. Summary of Characteristics for All Included Studies.

Study	Year	Cohort + visit	Visit	Median follow-up	Country	Total N	% women	HF events	Median age	Biomarkers	Covariates	Quality
AbouEzzeddine	2016	Olmsted County	1997-2000	11.0	USA	1824	53.1	173	61.0	NT-proBNP, hs-cTnI, BNP, Gal-3	1, 2, 5, 6, 12, 13, 14, 22	Good
Aguilar	2020	ARIC	Visit 5	4.6	USA	4829	41.5	217	74.0	Gal-3	1, 2, 3, 5, 6, 11, 13, 18	Good
Almahmoud	2018	MESA		12.1	USA	6542	53.3	291	62.0	FGF-23	1, 2, 3, 4, 6, 11, 13, 18	Good
Binnenmars	2022	PREVEND	Second survey (2002-2003)	7.4	The Netherlands	6830	50.3	227	53.8	FGF-23	1, 2, 5, 12, 13, 14, 16, 20, 21	Good
Brouwers	2014	PREVEND	1997-1998	12.5	The Netherlands	8569	50.2	374	49.0	NT-proBNP, hs-cTnT, MR-proANP, MR-proADM, Gal-3	1, 2, 5, 6, 7, 8, 11	Good

deFilippi	2010	Cardiovascular Health Study	Main cohort and supplemental black cohort	11.8	USA	4221	59.5	1279	73.0	cTnT	1, 2, 3, 6, 8, 10, 11, 13, 18, 22	Good
Djousse	2013	Cardiovascular Health Study	1989-1990 and 1992 - 1993	10.7	USA	4260	59.8	1182	75.0	FABP4	1, 2, 3, 4, 5, 11, 14, 15, 16	Good
Echouffo-Tcheugui	2022	ARIC	Visit 3	23.0	USA	10570	54.1	2429	60.0	GDF-15	1, 2, 3, 4, 5, 6, 8, 10, 11, 14, 15, 18, 19	Good
Fluschnik	2018	DAN-MONICA	Baseline cohort	27.4	Denmark	3785	48.7	-	45.5	GDF-15	1, 2, 6, 11, 13, 14	Good
Frary	2020	MONICA	1982-1994	18.5	Denmark	1951	49.9	-	54.0	NT-proBNP	1, 2, 6, 8, 11	Good
Funke-Kaiser	2014	FINRISK		14.0	Finland	7899	50.3	505	48.0	NT-proBNP, MR-proANP, BNP, MR-proADM	1, 4, 6, 8, 10, 11, 13, 18	Good
Ghorbani	2018	Framingham Heart Study Offspring	Offspring (1995-1998, 2005-2008)	7.8	USA	2477	54.9	108	57.0	Gal-3	1, 2, 5, 6, 11, 13, 14, 17, 18, 24	Good
Giuseppe	2014	EPIC-Potsdam cohort		8.2	Germany	1349	56.1	221	53.0	FGF-23	1, 2, 5, 11, 12, 13, 14, 15, 16, 22	Good

Harris	2010	Cardiovascular Health Study	Main cohort and supplemental black cohort	11.9	USA	4312	59.4	710	73.0	NT-proBNP	1, 2, 3, 5, 8, 10, 11, 12, 13, 14, 18, 22	Good
Ho	2018	Framingham Heart study	Offspring cohort and third generation cohort.	14.3	USA	3523	53.3	226	62.0	NT-proBNP, GDF-15, MPO, FGF-23, PAI-1, IGF-1, IGFBP-1, IGFBP-2	1, 2, 5, 6, 8, 10, 11, 13, 18, 20, 21	Good
Ho	2012	Framingham Heart Study	Sixth examination	11.2	USA	3353	53.1	166	59.0	Gal-3	1, 2, 5, 6, 11, 13, 18, 21, 22, 23	Good
Jagodzinski	2015	FINRISK		15.0	Finland	8312	49.2	641	48.8	Gal-3	1, 2, 4, 6, 8, 10, 11, 13, 14, 23	Good
Kaplan	2008	Cardiovascular Health Study	Main cohort and supplemental black cohort	6.2	USA	1638	62.1	566	74.3	IGF-1, IGFBP-1	1, 2, 3, 5, 6, 8, 9, 10, 12, 13, 14, 17, 22	Good
Lind	2022	PIVUS	2001-2004	15.0	Sweden	2449	50.0	132	75.0	NT-proBNP	2, 5, 6, 9, 10, 11, 13, 21	Good
Lutsey	2014	ARIC	Visit 2	18.6	USA	11638	57.5	1515	57.0	FGF-23	1, 2, 3, 5, 6, 9, 10, 11, 13, 15, 18, 19	Good

Magnussen	2019	DanMONICA, FINRISK, Moli-sani, Northern Sweden	Baseline examinations between 1982-2010	12.7	Denmark, Finland, Italy, Sweden	78657	51.7	5170	49.4	NT-proBNP	1, 2, 5, 6, 8, 11, 12, 13	Good
McEvoy	2016	ARIC	CARMRI substudy	6.8	USA	1809	51.0	167	71.0	Gal-3	1, 2, 4, 6, 8, 10, 11, 13, 14, 18	Good
McKie	2014	Olmsted County	1997-2000	10.7	USA	1843	52.0	193	62.0	hs-cTnI	1, 2, 5, 6, 11, 12, 13, 22	Good
Molvin	2020	Malmo Preventive Project		10.8	Sweden	1713	29.1	130	67.4	FABP4, PAI-1, IGFBP-2	1, 2, 5, 6, 11, 13, 18, 21, 24	Good
Nambi	2013	ARIC	Visit 4	10.4	USA	9868	55.7	970	62.7	NT-proBNP, hs-cTnT	1, 3, 5, 6, 11, 13, 18, 22	Good
Neumann	2014	FINRISK		14.0	Finland	7899	50.3	505	47.8	hs-cTnI	1, 4, 6, 8, 10, 11, 13, 18	Good
Nguyen	2020	MESA	Examination 1	12.4	USA	9868	55.7	970	62.7	hs-cTnT	1, 2, 3, 5, 9, 10, 11, 12, 14	Good
Oyenuga	2018	ARIC Carotid MRI study		9.6	USA	1465	57.8	163	70.0	MPO	1, 2, 3, 5, 6, 8, 10, 11, 13, 18, 19	Good

Paul	2021	CARDIA		7.1	USA	3152	56.0	33	45.2	FGF-23	1, 2, 3, 5, 11, 13, 14	Good
Saha	2022	Jackson Heart Study	Visit 1, 2	12.0	USA	2423	64.1	139	53.0	hs-cTnI	1, 2, 5, 6, 11, 12, 13, 17	Good
Segar	2021	ARIC	Visit 5	4.6	USA	5210	57.6	232	75.0	NT-proBNP	1, 2, 3, 5, 6, 10, 11, 12, 13, 14, 19, 24	Good
Sharma	2020	Cardiovascular Health Study		10.0	USA	844	61.5	220	78.0	FGF-23	1, 2, 3, 5, 6, 8, 11, 13, 14, 18	Fair
Smith	2010	Malmo Diet and Cancer Study	Baseline examination between 1991 and 1996	13.8	Sweden	5135	59.2	112	57.6	NT-proBNP, MR-proANP, MR-proADM	1, 2, 5, 6, 9, 10, 11, 13, 18, 20	Good
Stenemo	2018	PIVUS	2001-2004	10.0	Sweden	901	50.7	80	70.2	FABP4, GDF-15, FGF-23, Gal-3	1, 2	Good
		ULSAM	1998-2001	8.0	Sweden	685	0.0	90	77.8	FABP4, GDF-15, FGF-23, Gal-3	1, 2	Good
Suthahar	2020	Framingham Offspring Study, PREVEND, MESA,	Offspring cohort examination 6, PREVEND examination 1, MESA examination 1, and CHS examination 1	12.6	USA, the Netherlands	22756	53.1	2095	60.0	PAI-1	1, 5, 11, 12, 13, 17, 20, 21	Good

		Cardiovascular Health Study										
Suthahar	2021	PREVEND	Baseline 1997-1998	11.3	The Netherlands	8202	50.0	357	49.2	cTnT, PAI-1	1, 2, 5, 8, 11, 12, 13, 14, 20, 21	Good
Tang	2021	ARIC	Visit 5	6.3	USA	5876	58.0	206	75.5	hs-cTnI	1, 2, 3, 6, 8, 10, 11, 13, 18, 19	Good
Tang	2009	Cardiovascular Health Study		7.2	USA	3733	61.3	569	75.0	MPO	1, 2, 6, 9, 11, 13, 20, 24	Good
Velageleti	2010	Framingham Offspring Study	Offspring sixth examination	9.4	USA	2754	53.6	95	58.0	BNP	1, 2, 5, 6, 11, 13, 18, 20, 23	Good
Wang	2012	Framingham Offspring Study	Offspring sixth examination	11.3	USA	3428	53.1	162	59.0	hs-cTnI, GDF-15	1, 2, 5, 6, 8, 10, 11, 13, 17, 18, 21, 24	Good
Wannamethee	2014	British Regional Heart Study		11.0	United Kingdom	3672	0.00	257	69.0	NT-proBNP	1, 6, 11, 14, 15, 16, 17, 18, 20	Good
Welsh (1)	2019	Generation Scotland Scottish		7.8	Scotland	19285	58.5	216	46.9	hs-cTnI, hs-cTnT	1, 2, 6, 8, 10, 11, 13, 18, 19	Good

		Family Health Study										
Welsh (2)	2019	British Regional Heart Study		12.6	United Kingdom	3852	0	295	68.0	hs-cTnT	1, 5, 6, 7, 8, 10, 11, 13, 14, 15, 16, 18, 19, 20, 21	Good
Wu	2022	ViDA		5.0	New Zealand	4102	45.1	248	65.2	NT-proBNP, hs-cTnI, hs-cTnT	1, 2, 3, 6, 11, 13, 18, 19, 21	Good
		HVOLS		5.0	New Zealand	2528	32.6	227	64.3	NT-proBNP, hs-cTnI, hs-cTnT	1, 2, 3, 6, 11, 13, 18, 19, 22	Good
Yan	2020	DanMONICA, FINRISK, Moli-sani, MONICA	DanMONICA: 1982-1992. FINRISK: 1997. Moli-sani: 2005-2010. Northern Sweden MONICA: 1986-2009.	13.8	Denmark, Finland, Italy, Sweden	48455	51.9	1990	50.7	NT-proBNP, hs-cTnI	1, 2, 5, 6, 9, 11, 13, 14, 18	Good
Zhu (1)	2018	Busselton Health Study		20.0	Australia	3215	57.2	121	50.0	hs-cTnI	1, 2, 6, 7, 8, 10, 11, 13, 14, 16, 18	Good
Zhu (2)	2022	Rotterdam Study	RS-I-3 and RS-II-1	13.6	The Netherlands	5430	59.9	664	68.6	hs-cTnT	1, 6, 8, 10, 11, 13, 18	Good

Covariates: age[1], sex[2], ethnicity[3], clinic site[4], body mass index[5], systolic blood pressure[6], plasma glucose[7], total cholesterol[8], low-density lipoprotein cholesterol[9], high-density lipoprotein cholesterol[10], smoking status[11], hypertension[12], diabetes mellitus[13], renal function[14], physical activity[15], alcohol intake[16], left ventricular hypertrophy[17], anti-hypertensive medication[18], cholesterol lowering-medication[19], history of myocardial infarction[20], history of atrial fibrillation[21], history of coronary artery disease[22], history of valvular disease[23], history of cardiovascular disease[24].

BNP, b-type natriuretic peptide; FABP4, fatty acid binding protein 4; FGF-23, fibroblast growth factor 23; HF, heart failure; Gal-3, galectin-3; GDF-15, growth differentiation factor 15; hs-cTnI, high-sensitivity cardiac troponin I; hs-cTnT, high-sensitivity cardiac troponin T; MPO, myeloperoxidase; MR-proADM, midregional proadrenomedullin; MR-proANP, midregional pro-atrial natriuretic peptide; N, number; NT-proBNP, N-terminal pro b-type natriuretic peptide; PAI-1, plasminogen activator inhibitor-1.

Table A.2. Biomarker Associations with Incident Heart Failure, Stratified by HFrEF and HFpEF.

**Biomarker**	**Author**		**HFrEF**	**HFpEF**
NT-proBNP	Brouwers	Low risk group	1.77 (1.27-2.47)	1.30 (0.87-1.96)
		High risk group	3.22 (2.45-4.23)	1.69 (1.25-2.27)
	Segar		9.60 (4.36-21.11)	4.70 (2.55-8.66)
hs-cTnI	Saha		2.15 (1.48-3.11)	2.18 (1.56-3.04)
hs-cTnT	Brouwers	Low risk group	1.50 (1.16-1.94)	1.12 (0.85-1.46)
		High risk group	1.78 (1.52-2.07)	1.41 (1.10-1.79)
MR-proANP	Brouwers	Low risk group	1.05 (0.74-1.47)	1.09 (0.78-1.53)
		High risk group	2.96 (2.07-4.21)	1.49 (0.92-2.40)
MR-proADM	Brouwers	Low risk group	0.83 (0.59-1.17)	0.71 (0.47-1.08)
		High risk group	1.52 (1.32-1.77)	1.24 (0.86-1.80)
FGF-23	Almahmoud		1.13 (0.93-1.37)	1.31 (1.11-1.55)
	Binnenmars		1.33 (1.06-1.68)	1.25 (0.91-1.70)
Galectin-3	Brouwers	Low risk group	0.94 (0.71-1.23)	0.82 (0.48-1.39)
		High risk group	1.46 (1.26-1.70)	0.92 (0.70-1.22)
	Ghorbani		1.42 (1.02–1.96)	1.39 (1.05–1.84)

FGF-23, fibroblast growth factor 23; HF, heart failure; HR, hazard ratio; hs-cTnI, high-sensitivity cardiac troponin I; hs-cTnT, high-sensitivity cardiac troponin T; MR-proADM, midregional proadrenomedullin; MR-proANP, midregional pro-atrial natriuretic peptide; NT-proBNP, N-terminal pro b-type natriuretic peptide.

Table A.3. Subgroup Analysis of Combining Natriuretic Peptides and Troponins in Model Adjustments.

Base biomarker	Author	Base model	Addition of biomarker	Additional adjustment
NT-proBNP	AbouEzzeddine	2.00 (1.62-2.46)	1.75 (1.42-2.16)	hs-cTnI
	Brouwers	2.17 (0.96-4.92)	5.54 (2.75-11.16)	hs-cTnT
	Nguyen	2.56 (2.20-2.98)	2.09 (1.80-2.43)	hs-cTnT
Hs-cTnI	AbouEzzeddine	1.66 (1.44-1.90)	1.52 (1.31-1.75)	NT-proBNP
	Neumann	1.68 (1.04-2.72)	1.11 (1.01-1.21)	NT-proBNP
	McKie	1.33 (1.04-1.69)	1.42 (1.20-1.68)	NT-proBNP
Hs-cTnT	Brouwers	1.94 (0.88-4.23)	5.54 (2.75-11.16)	NT-proBNP
	Nguyen	1.89 (1.71-2.08)	1.66 (1.50-1.84)	NT-proBNP
	Welsh[2]	1.58 (1.42-1.77)	1.34 (1.19-1.52)	NT-proBNP
BNP	AbouEzzeddine	1.56 (1.33-1.84)	1.46 (1.24-1.72)	hs-cTnI

BNP, b-type natriuretic peptide; hs-cTnI, high-sensitivity cardiac troponin I; hs-cTnT, high-sensitivity cardiac troponin T; NT-proBNP, N-terminal pro b-type natriuretic peptide.
